# Identification of common and divergent gene expression signatures in patients with venous and arterial thrombosis using data from public repositories

**DOI:** 10.1371/journal.pone.0235501

**Published:** 2020-08-11

**Authors:** Bidossessi Wilfried Hounkpe, Rafaela de Oliveira Benatti, Benilton de Sá Carvalho, Erich Vinicius De Paula

**Affiliations:** 1 School of Medical Sciences, University of Campinas, Campinas, SP, Brazil; 2 Department of Statistics, Institute of Mathematics, Statistics and Scientific Computing, University of Campinas, Campinas, SP, Brazil; 3 Hematology and Hemotherapy Center, University of Campinas, Campinas, SP, Brazil; Medical University Innsbruck, AUSTRIA

## Abstract

Cardiovascular disease (CVD) and venous thromboembolism (VTE) figure among the main causes of morbidity and mortality in modern societies. Although associated with distinct pathogenic mechanisms, epidemiological, experimental and clinical trial data suggest that the mechanisms responsible for arterial and venous thrombosis are at least partially overlapped. Herein we aimed to explore shared and discordant pathways involved in the pathogenesis of VTE and CVD at the transcriptomic level and to validate the results in independent cohorts. Five public datasets of gene expression data from VTE and CVD (myocardial infarction, peripheral arterial occlusive disease and stroke) patients were analyzed using an integrative bioinformatic strategy. A machine/statistical learning method was used to derive classifiers for the discrimination of VTE and CVD, and tested in independent datasets. Two sets of genes that were commonly (n = 472) or divergently (n = 124) expressed in CVD and VTE were identified. Genes and pathways associated with innate immune function were over-represented in both conditions, along with pathways associated with complement and hemostasis. Pathways associated with neutrophil activation and with IL-1 signaling were also enriched in CVD compared to VTE. The gene expression signature of VTE more closely resembled the pattern of cardioembolic stroke than the patterns of acute myocardial infarction, ischemic stroke and peripheral arterial occlusive disease. Classifiers derived from these gene lists accurately discriminated patients with VTE and CVD from independent cohorts. In conclusion, our results add a new set of data at the transcriptomic level for future studies between arterial and venous thrombosis.

**Strengths and limitations of this study:**

- Our results represent the first comparison of venous and arterial thrombosis at the transcriptomic level.
- Our main result was the demonstration that immunothrombosis pathways are important to the pathophysiology of these conditions, also at the transcriptomic level.
- A specific signature for venous and arterial thrombosis was described, and validated in independent cohorts.
- The limited number of public repositories with gene expression data from patients with venous thromboembolism limits the representation of these patients in our analyses.
- In order to gather a meaningful number of studies with gene expression data we had to include patients in different time-points since the index thrombotic event, which might have increased the heterogeneity of our population.

## Introduction

CVD is a generical term that encompasses conditions caused by arterial thrombosis such as myocardial infarction (MI), ischemic stroke (IS) and peripheral arterial obstructive disease (PAOD), with the former two representing the most frequent causes of years of life lost in most regions of the world [1, 2]. Venous thromboembolism (VTE) encompasses deep vein thrombosis (DVT) and pulmonary embolism (PE), which together represent the third leading cause of vascular disease in the world [3]. Although it has been long recognized that the pathogenesis of these two conditions are based on distinct cellular and molecular pathways, the existence of common pathogenic pathways contributing to both CVD and VTE is suggested by (i) the sharing of risk factors such as obesity, smoking, hypertriglyceridemia [4]; (ii) the epidemiological association between CVD and VTE illustrated by the higher prevalence of CVD in patients with VTE even years after the venous event [5–7]; (iii) the fact that some inflammatory diseases such as sickle cell disease and antiphospholipid syndrome (APS) increase the risk of both conditions [8, 9]; and, (iv) more recently, the demonstration that treatment strategies classically used for CVD can also benefit patients with VTE [10, 11], and vice versa [12]. In this context, a lot remains to be learned about their shared and independent pathological mechanisms, whose identification could contribute to the identification of new therapeutic targets for both VTE and CVD [7, 13, 14].

Three major frameworks have been used to address differences and similarities between CVD and VTE: (i) studies in animal models, (ii) histopathological analyses of thrombi, and (iii) epidemiological data. Studies in animal models identified proteins and cells that contribute to VTE or CVD [2, 15–17] allowing the development of important therapeutic targets for each condition. However, these studies have not focused on the relative contribution of these pathways to CVD, VTE or both conditions in human disease. While histopathological studies of human thrombin initially supported the classical paradigm of white (platelet-rich) or red (fibrin- and red blood cell-rich) thrombi in CVD and VTE respectively, these conclusions were later challenged by several studies showing a much more complex picture, as recently reviewed [13]. Lastly, epidemiological studies have been instrumental to gain insights into the association of venous and arterial thrombosis, and clearly demonstrated that VTE and CVD are indeed associated conditions [18, 19]. However, these studies have not been yet able to clearly define the mechanism of this association, whether causal (i.e. atherosclerosis leads to VTE) or driven by common pathogenic mechanisms [7].

In recent years, the availability of large databases of genomic data, along with bioinformatics and machine learning tools capable of performing integrative and functional analyses of these datasets allowed new strategies for the research about the molecular and cellular pathogenesis of complex conditions. In particular, publicly available datasets from gene expression studies, once performed to define specific disease signatures, can now be compared, grouped and meta-analyzed, allowing biases and artefacts to be canceled out between datasets, so that true relationships are more likely to stand out [20–26]. Herein we used a panel of bioinformatics and machine learning tools to explore the differences and similarities between VTE and CVD, thus contributing with a new layer of data to our understanding of the common and divergent pathogenic mechanisms of two conditions of high epidemiological relevance.

## Methods

### Identification of eligible studies and datasets

Gene expression datasets from microarray studies including human patients with CVD or VTE were searched in the public repository Gene Expression Omnibus (GEO) [27], maintained by the NCBI, by May 2018. Search was conducted using the terms “venous thrombosis”, “venous thromboembolism”, “myocardial infarction”, “stroke”, “coronary ischemia”, “angina”, “atherosclerosis”, “peripheral arterial disease” or “thrombosis”. Datasets were included if they met all the following inclusion criteria: (1) microarray data obtained from human samples using the same microarray platform; (2) RNA source restricted to whole blood or populations of circulating blood cells; (3) studies including both affected patients and healthy controls, so that the differential expression of each gene was evaluated under the same experimental conditions; (4) availability of metadata allowing the separation of venous from arterial events; and (5) datasets from studies published in peer-reviewed journals. In the course of our study, we also restricted our analysis to studies using the same microarray platform, so as to limit heterogeneity.

### Patient and public involvement

No patient involved.

### Meta-analysis of gene expression studies

#### Pre-processing

Microarray raw data were pre-processed using the *Robust Multichip Average* (RMA) method [28] implemented in the *oligo* package [29]. For each dataset, the algorithm performs background subtraction, minimizing the effects of optical noise and non-specific binding on the estimation of relative gene expression parameters. Later, quantile normalization was applied, mitigating the effects of technical variables through the estimation of a common intensity distribution across samples. This stage was followed by a median-polish step, which summarized the several probe intensity measurements into a single probeset log-expression quantity, for the downstream meta-analysis step. Using the *biomaRt* package [30], we annotated the probesets with their respective Ensembl Gene IDs.

#### Meta-analysis

To perform the meta-analysis, expression data were organized following their pre-defined classes and study of origin. Meta-analysis was performed with *RankProd* package [31]. The algorithm of this package adapts the rank production method initially designed to single experiment analysis to integrate multiple origin studies. It is a non-parametric method that detects genes consistently ranked as DE by comparing patients to healthy controls across datasets. One hundred permutations were performed to compute the p-value and the false discovery rate (FDR). The gene list was further filtered to include only genes that were up- or down-regulated in the same direction in all five studies based on a false discovery rate (FDR) < 0.05.

### Correlation analysis of gene expression levels in CVD and VTE

The correlation between the expression levels of genes identified in the meta-analysis between VTE and CVD was expressed using the estimated Pearson’s coefficient, and then represented in graphical format. Unless otherwise stated, all analyses were performed in the statistical computing environment R version 3.4.4 [32].

### Functional gene set analyses

To facilitate the interpretation of the biological significance of the gene list identified by the meta-analysis, a functional gene set analysis (GSA) was performed using EnrichR, a bioinformatics web-based tool that includes several curated GSA libraries encompassing pathway enrichment analysis (e.g. KEGG, Reactome, and 18 other libraries), gene ontologies (for cellular components, biological process, molecular function), among others. Of the list of enriched terms identified by EnrichR, only pathways that were (i) listed among the 10 most significant (based on the p-value) for each library, and (ii) identified in at least two libraries from the same category were considered. For gene ontology terms, the top 5 terms with an adjusted p-value < 0.0001 were included.

### Evaluation of genes with divergent expression between VTE and CVD

A list of genes with divergent expression between VTE and three databases of CVD (IS, AMI and PAOD) was obtained by selecting all genes with a fold-change higher than 1.5 that were up-regulated in VTE and down-regulated in IS, AMI and PAOD; as well as genes with a fold-change lower than 0.8 that were down-regulated in VTE and up-regulated in IS, AMI and PAOD. The cutoff values are defined as the percentile 25% (0.8) and 75% (1.49~1.5) fold change to prioritize the most down and up-regulated genes respectively. Similar filtering approach has been used to avoid the definition of arbitrary threshold [33–35].

These gene lists were used for an additional functional analysis based on FAIME (Functional Analysis of Individual Microarray/RNAseq Expression) scores. The FAIME algorithm is implemented in *seq2pathway* package [36] and computes the cumulative quantitative effects of genes inside differentiated Gene Ontology terms using log2 gene expression of each individual sample. The result was clustered based on their gene pattern similarities using Euclidean distances and plotted in a FAIME score heat map.

### Validation of gene expression signatures associated with VTE and CVD

In order to validate our results in independent cohorts, we first used Support Vector Machine (SVM) based methods to identify two subsets of genes capable to more accurately separate VTE from CVD (validation 1) and VTE (validation 2) as well as AMI and IS (validation 3 and 4) from controls. SVM-based models are based on statistical learning theory [37], and are normally used to optimize the discriminatory power of complex datasets by identifying subsets of data with higher discriminatory potential (classifiers) [38, 39]. For validation 1, SVM was applied to the list of genes that were divergently expressed between VTE and CVD, using the VTE and AMI patients’ datasets employed for our meta-analysis as training cohorts. The list of classifiers was then tested in three additional cohorts (validation cohorts) that were not used in the meta-analysis, constituted of patients with VTE (GSE48000) [40], AMI (GSE59867) [41]. For validation 2, the training cohort consisted of the dataset of VTE patients used in our meta-analysis (GSE19151), and the validation cohort consisted of a different dataset of VTE patients (GSE48000) [40]. Finally a training cohort for validation 3 and 4 consisted healthy controls and patients of the AMI and IS datasets (GSE59867, GSE22255 respectively). Results were then validated using the cohorts consisted of another AMI and IS datasets (GSE141512 and GSE16561 respectively) and presented as heat map [42] of normalized expression.

## Results

### Studies included in the meta-analysis

Five studies fulfilled the inclusion and exclusion criteria described in methods section, and were included in the meta-analysis. These studies included data from 163 adult patients and 145 healthy controls. Table 1 provides the details of each study. As shown in Table 1, only one study include patients with VTE and compared gene expression levels in patients with single or recurrent VTE (GSE19151) with healthy controls [43]. The other four remaining studies involve CVD. These CVD studies present gene expression levels of patients with PAOD (GSE27034) [44], AMI (GSE48060) [45], cardioembolic stroke (GSE58294) [46], and IS (GSE22255) [47]. All of them have appropriated study-specific paired healthy controls.

**Table 1:** Characteristics of individual studies included in our analyses.

GEO access number	Sample characteristics		
	Characteristics of patients/disease included in each dataset	Size (Pt:Ctl)	RNA source
GSE19151	Adult with one or more prior VTE or warfarin; APS and cancer excluded	70:63	Whole blood
GSE27034	Peripheral arterial occlusive disease, defined as ankle:brachial index < 0.9	19:18	PBMC
GSE48060	Adults with 1^st^ time acute myocardial infarction*; inflammatory diseases and cancer excluded.	31:21	Whole blood
GSE58294	Adults with cardioembolic stroke (i.e. at least one source of cardiac embolus and exclusion of strokes from other etiologies) †	23:23	Whole blood
GSE22255	Adults with history of one ischemic stroke more than 6 months prior to sample collection; anemia and allergies excluded	20:20	PBMC

GEO: Gene Expression Omnibus, Pt:Ctl: patients:controls; PBMC: peripheral blood mononuclear cells; WBC: white blood cells; VTE: Venous thromboembolism; APS: antiphospholipid syndrome.

* Samples were collected with 48h from the acute event

† subset of patients recruited for the Clear Stroke Trial [48]; samples were collected within 3h from the acute event, prior to any pharmacological treatment. All studies used the Affymetrix Human Genome U133 Plus 2.0 as a microarray platform. References for published studies that used these datasets are indicated in the main text.

### Similarities in gene expression profiles of arterial and venous thrombosis

The meta-analysis of all studies identified 168 up-regulated and 304 down-regulated DE genes (S1 Table). The top 10 up- and down-regulated genes are shown in Table 2. Since the four studies of CVD included patients in the acute or chronic phases of their disease courses, we also present separate meta-analyses of acute (AMI and CS) and chronic (IS and PAOD) CVD (S2 and S3 Tables).

**Table 2:** Top differentially expressed genes identified in the meta-analysis.

	Fold-change in individual studies (FC)						Meta-analysis results		Main biological process
Genes	VTE	PAOD		AMI	CS	IS	Ave FC	FDR	
**Up-regulated genes**									
*G0S2*	1.20		4.88	3.89	1.12	1.76	2.57	<0.0001	Apoptosis
*BCL2A1*	2.29		1.55	1.73	1.30	1.88	1.75	<0.0001	Apoptosis
*TNFAIP6*	2.05		1.45	1.72	1.23	1.70	1.63	<0.0001	Innate Imm
*ANXA3*	1.71		1.40	1.21	1.83	1.96	1.62	<0.0001	Hemostasis
*SERPINB2*	1.19		1.91	1.42	1.23	1.95	1.54	<0.0001	Hemostasis
*S100A12*	2.10		1.00	1.01	1.74	1.79	1.53	<0.0001	Innate Imm
*SLPI*	1.84		1.14	1.00	1.59	1.65	1.45	<0.0001	Innate Imm
*FKBP1B*	2.05		1.19	1.23	1.09	1.61	1.43	<0.0001	Imm Reg
*DEFA4*	1.25		1.55	1.27	1.01	2.08	1.43	<0.0001	Innate Imm
*PTX3*	1.06		1.98	1.56	1.11	1.35	1.41	<0.0001	Innate Imm
**Down-regulated genes**									
*CLIC3*	0.89		0.77	0.89	0.87	0.92	0.87	<0.0001	Cell Maint
*BACH2*	0.92		0.91	0.79	0.89	0.96	0.90	<0.0001	Imm Reg
*TXK*	0.85		0.94	0.93	0.87	0.63	0.84	<0.0001	Innate Imm
*MLC1*	0.87		0.97	0.98	0.94	0.97	0.95	<0.0001	Unknown
*ID3*	0.94		0.92	0.90	0.84	0.97	0.91	<0.0001	Cell Prolif
*ZNF304*	0.87		0.98	0.91	0.97	0.61	0.87	<0.0001	Gene expr
*EVL*	0.97		0.95	0.92	0.87	0.86	0.92	<0.0001	Innate Imm
*BCOR*	0.93		0.96	0.92	0.95	0.92	0.94	<0.0001	Apoptosis
*TBX21*	0.98		0.96	0.79	0.97	0.79	0.90	<0.0001	T cell dev
*IL2RB*	0.87		0.95	0.92	0.94	0.87	0.91	<0.0001	Imm Reg

Genes were ranked according to the fold change. FC: Fold-change; Ave FC: average FC; FDR: False Discovery Rate. VTE: venous thromboembolism; PAOD: peripheral arterial obstructive disease; AMI: acute myocardial infarction; CS: cardioembolic stroke; IS: ischemic stroke; Innate Imm: innate immunity; Imm Reg: Immune regulation; Cell Maint: cell maintenance; Cell Prolif: cell proliferation; Gene expr: gene expression; T cell dev: T cell development. The average FC is expressed as mean FC across studies.

**Fig 1:**
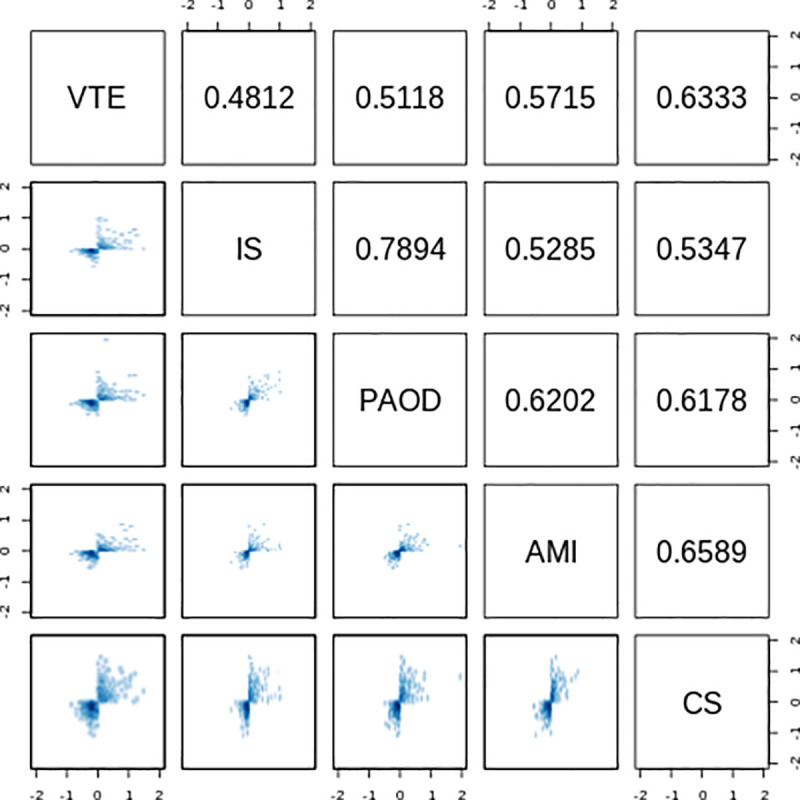
Correlation of gene expression changes between Venous Thromboembolism (VTE). Ischemic stroke (IS). Peripheral arterial occlusive disease (PAOD). Acute myocardial infarction (AMI) and Cardioembolic stroke (CS). Pairwise correlation scatter plots are in the lower triangle boxes. The upper triangle boxes show Pearson correlation coefficients (R) of log2 fold changes for all 472 differentially expressed genes between studies.

Next, we assessed the correlation of gene expression levels across all five studies using all 472 DE genes. As shown in Fig 1. VTE presented numerically lower correlation coefficient with IS and PAOD than those observed between all studies involving arterial thrombosis. We also evaluated the correlation of VTE with studies of CVD that included patients in acute and chronic phases separately (S1 Fig).

**Fig 2:**
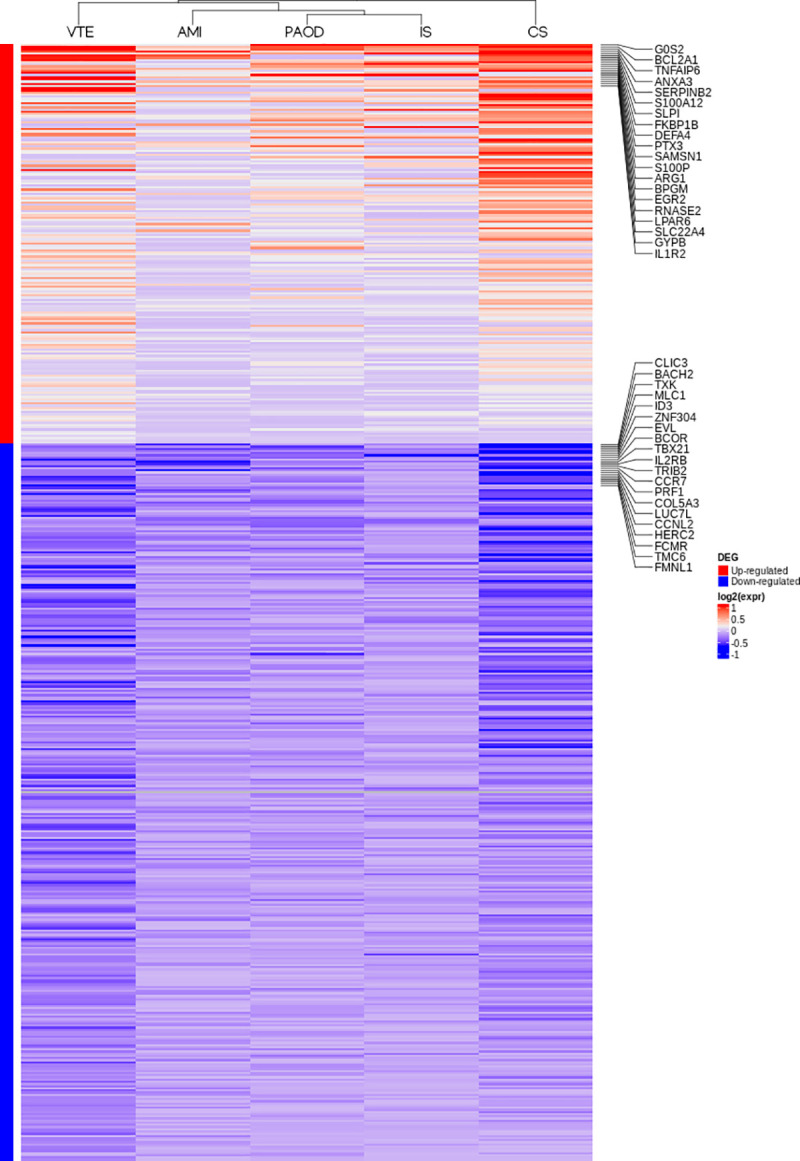
Fold-change of gene expression from patients with venous thromboembolism (VTE). Ischemic stroke (IS). Peripheral arterial occlusive disease (PAOD). Acute myocardial infarction (AMI). Cardioembolic stroke (CS). The top 20 up- (red) and down-regulated (blue) genes are listed.

Based on the lower correlation of gene expression changes between VTE and CVD we interrogated whether an unsupervised cluster analysis using the fold-change of the 472 genes identified in the meta-analysis could provide additional information on differences and similarities between VTE and CVD at a transcriptomic level. As shown in Fig 2 AMI, PAOD and IS were clustered together while the pattern of VTE was closer to CS than to this cluster (AMI. PAOD and IS).

### The shared gene expression signature between VTE and CVD is markedly associated with innate immunity

To evaluate which pathways and biological processes were associated with the gene expression signature shared between VTE and CVD (472 DEG) we performed a functional analysis using stringent criteria for the call of potentially relevant pathways and gene ontology terms. As shown in Table 3, pathways associated with hemostasis and innate immunity were consistently identified in these analyses.

**Table 3:** Pathways and ontology terms enriched in the meta-analysis of VTE and CVD.

Functional category	GSA library	*p-value*
*Pathways associated with hemostasis*		
Complement and coagulation cascades	KEGG	0.001
Blood clotting cascade	Wikipathways	0.001
Hemostasis	Reactome	0.0006
Intrinsic Prothrombin Activation Pathway	Biocarta	0.02
*Pathways/terms associated with activation of the immune system*		
IL-1 Signaling Pathway	Wikipathways	0.006
Signal transduction through IL1R	Biocarta	0.04
Immune System	Reactome	< 0.001
Adaptive Immune System	Reactome	< 0.001
Neutrophil degranulation	GO biological process	< 0.001
Neutrophil mediated immunity	GO Biological process	< 0.001
Neutrophil activation in immune response	GO Biological process	< 0.001
Antibacterial humoral response	GO Biological process	< 0.001
Innate immune response in mucosa	GO Biological process	< 0.001

Only pathways identified in more than one GSA library are listed (manually clustered according to common biological processes). For gene ontology terms the top five terms with an adjusted P value < 0.0001 were considered relevant. GSA: gene set analysis.

### Evaluation of genes that were divergently expressed between VTE and CVD

**Fig 3:**
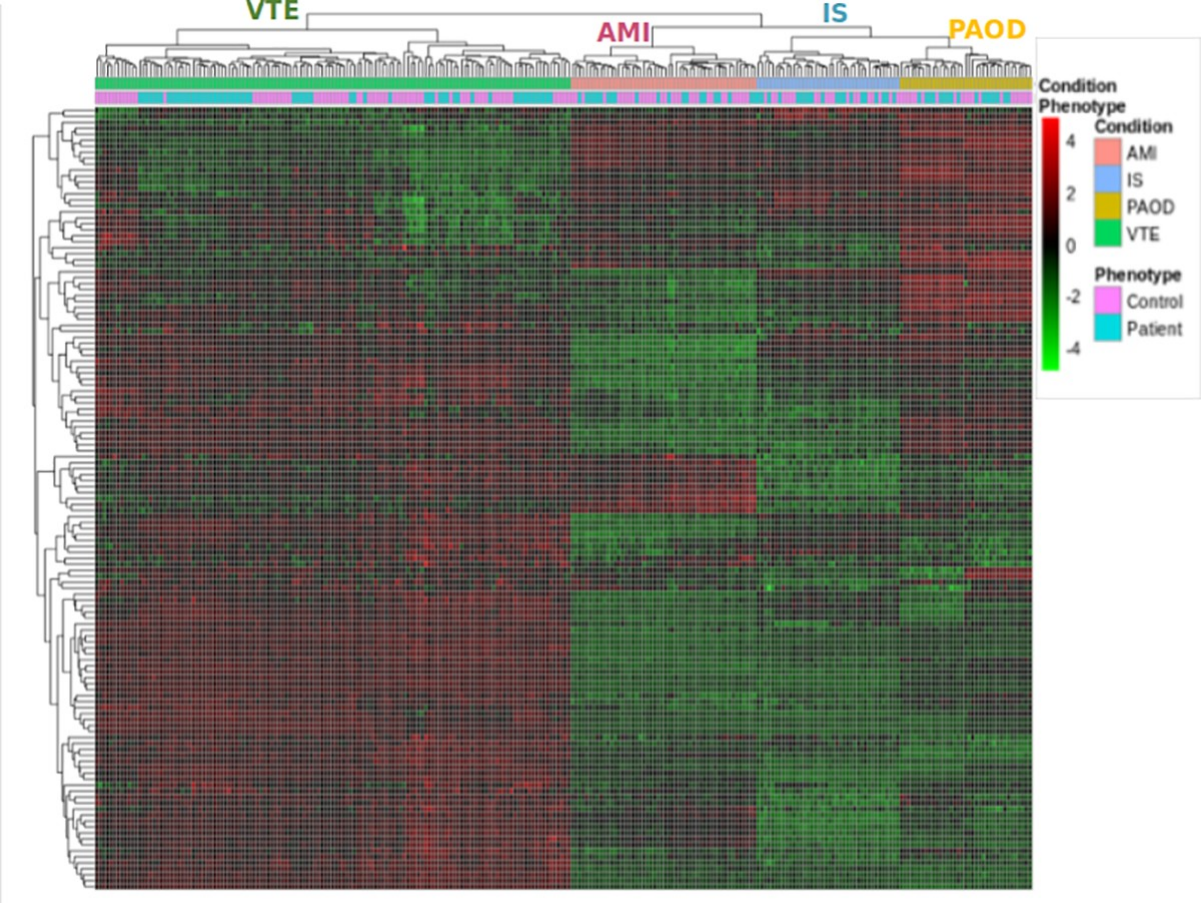
Heatmap built with the 124 DE genes that were divergently expressed between VTE and CVD. Each column represents an individual (control or patient) and conditions are shown in the upper row. Unsupervised clustering of these genes demonstrates that IS. MI and PAOD are clustered together, separately from VTE.

While the meta-analysis allowed us to gain insights on the similarities of VTE and CVD, we also wanted to identify the most relevant differences between these two conditions at the gene expression level. In order to do so we obtained a list of all genes whose direction of expression were divergent between venous and arterial thrombosis. In this analysis, CS–which clustered with VTE in the similarity analysis—was not included, so as to sensitize the analysis for differences between venous and arterial thrombosis. In total 124 genes were identified, of which 71 were up-regulated in VTE and down-regulated in CVD, and 53 were up-regulated in CVD and down-regulated in VTE (S4 Table). Expression levels of these genes are shown in Fig 3 which clearly demonstrates a different profile between VTE and CVD.

**Fig 4:**
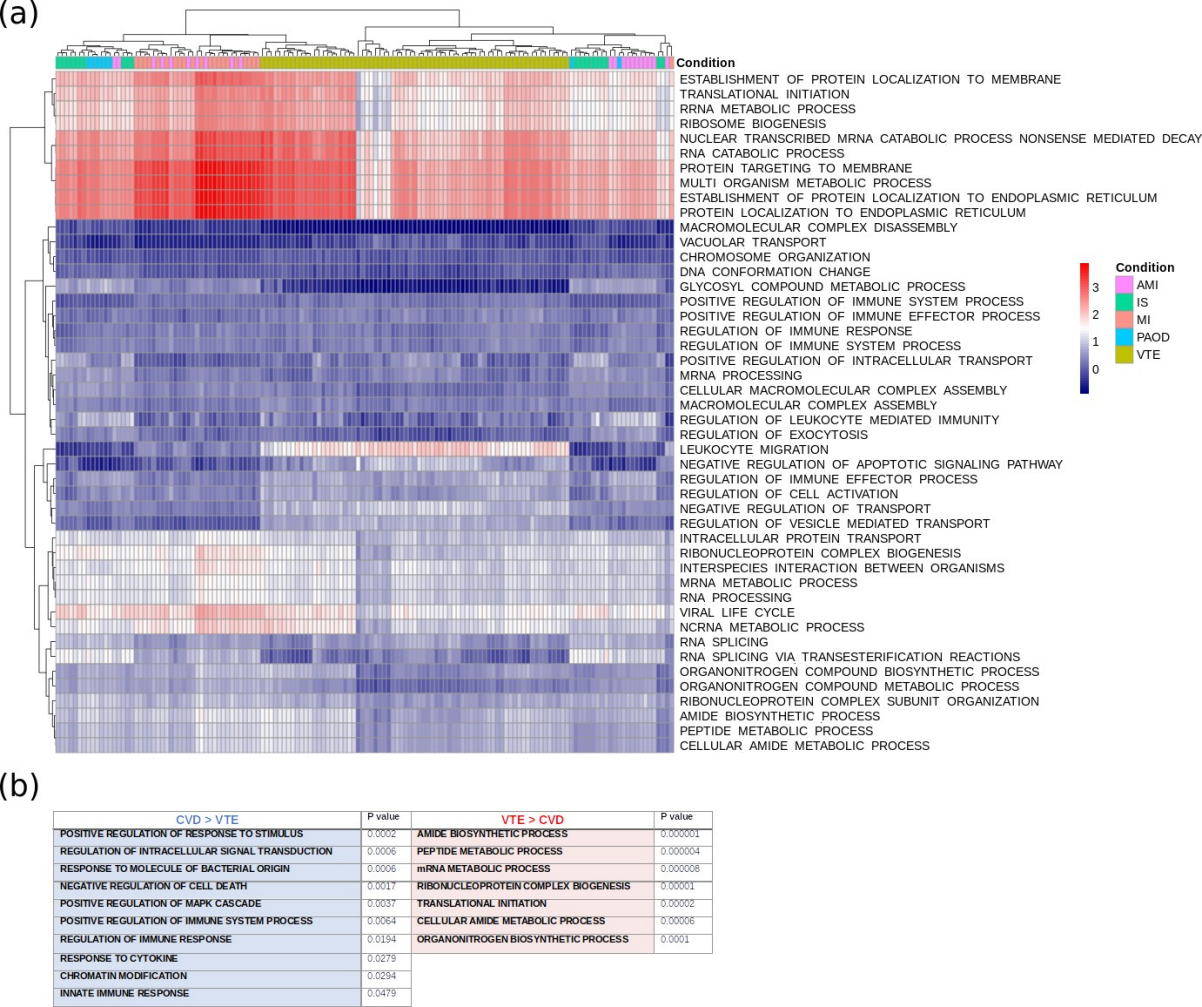
Heatmap (a) depicting FAIME scores of gene ontology (GO) terms enriched between VTE and CVD. FAIME scores compare the contribution of genes in different Gene Ontology pathways. Patients were segregated into VTE and CVD clusters based on their gene pattern similarities. The most significant terms were selected based on FDR (<0.05) and are summarized in the lower panel (b). VTE: venous thromboembolism; CVD: cardiovascular disease.

We then identified which pathways were associated with the expression signatures of these 124 genes. As shown in Fig 4A, genes that were up-regulated in VTE compared to CVD associated mainly with biological processes related to cell maintenance, cell proliferation and immune regulation. In contrast, genes that were up-regulated in CVD compared to VTE were associated mainly with innate immunity, neutrophil degranulation and cell proliferation (Fig 4B).

### Validation of gene expression signatures associated with VTE and/or CVD

**Fig 5:**
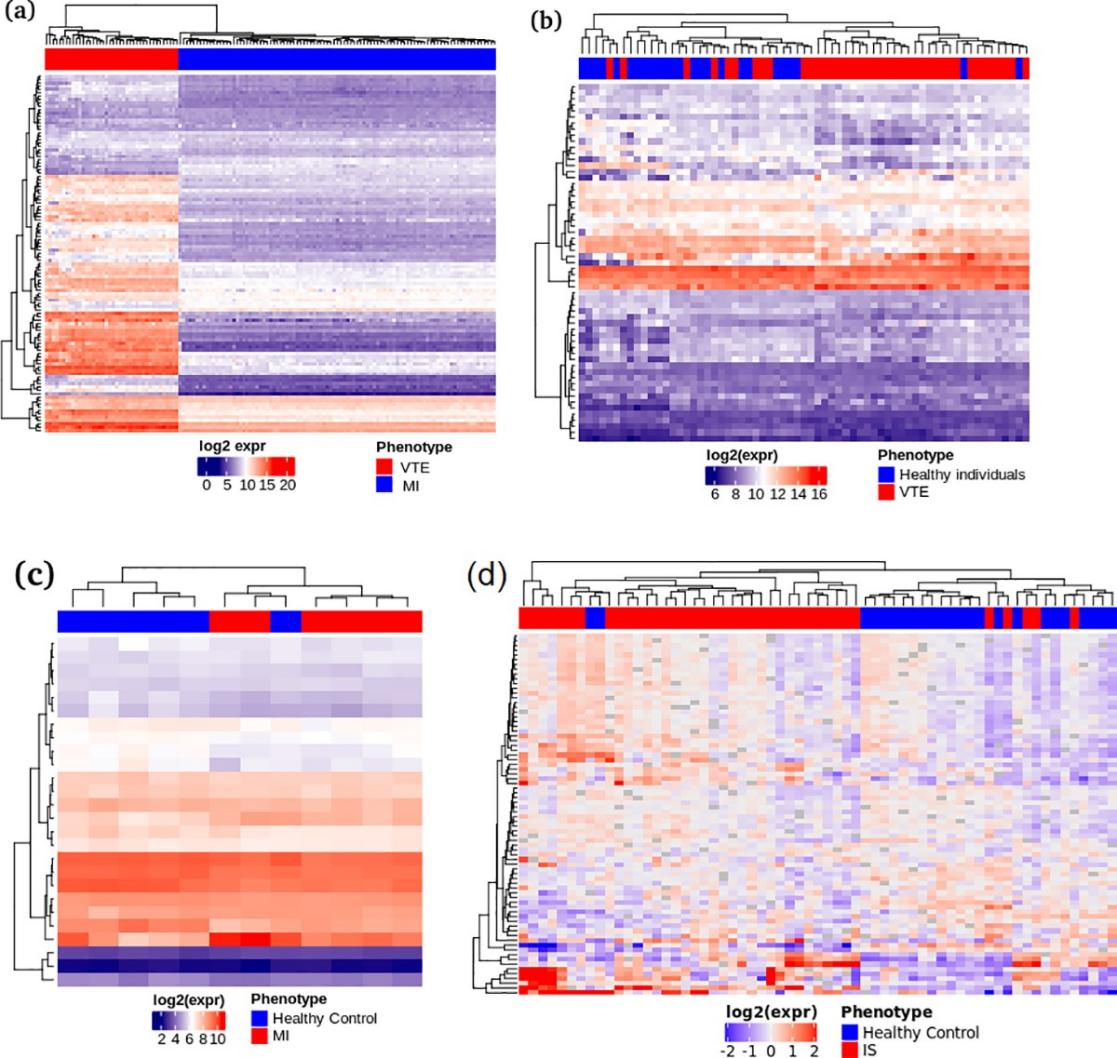
Clustering of VTE and CVD patients form independent (validation) cohorts, using gene lists (classifiers) identified by SVM-based methods derived from cohorts (training) used for the meta-analysis. In (a), a classifier consisting of 107 genes was capable to discriminate patients with at least two episodes of VTE (n = 71; red) from patients with AMI (n = 30; blue) with 100% accuracy. In (b), a classifier consisting of 60 genes was capable to discriminate the same population of VTE patients from healthy individuals with 76.5% accuracy. In (c), a classifier consisting of 23 genes was capable to discriminate the myocardial infarction patients from healthy individuals with 91.6% accuracy. Finally, (d) showed a classifier consisting of 76 genes capable to discriminate ischemic stroke patients from healthy individuals with 81.4% accuracy. log2(expr): base 2 logarithm of normalized expression; MI: myocardial infarction; VTE: venous thromboembolism; SVM: Support Vector Machine.

Finally, we validated the biological relevance of these gene expression signatures by constructing three SVM-based gene lists (classifiers) including one of the most informative genes from the list of divergently expressed genes (n = 124), and two from the commonly expressed genes (n = 472) (validation 1, 2, 3 and 4, respectively). The classifiers are presented in S5 Table. In validation 1, we were able to demonstrate that a classifier consisting of 107 genes could discriminate patients with at least two episodes of non-provoked VTE from patients with AMI with 100% accuracy (Fig 5A). We also show that a 60-gene classifier (validation 2) and a 23-gene classifier could discriminate patients with VTE and those with AMI from healthy individuals with an accuracy of 76.5% (Fig 5B) and 91.6% (Fig 5C) respectively. A classifier based on a gene set constituted of 76 commonly expressed genes were also capable to discriminate IS patients from healthy controls with 81.4% of precision (Fig 5D).

## Discussion

While both CVD and VTE are caused by the formation of thrombi inside a vessel, differences in their pathogenesis have been long recognized, with CVD linked to atherosclerosis [49] and VTE to the classical elements of the Virchow’s Triad [2, 50]. Yet, epidemiological, pathological and clinical data highlight the need for studies addressing in more detail the similarities and differences, particularly at the cellular and molecular level, between these two conditions. By using an integrative bioinformatics approach we were able to confirm that innate immunity, complement activation and classical hemostasis pathways are involved in the pathogenesis of both CVD and VTE at the transcriptomic level. In addition, we identified a shared and a discordant gene expression signature from VTE and CVD patients that can be used by other groups for the identification of biomarkers and therapeutic targets, as well as for a better understanding of the pathophysiology of these conditions.

VTE is a disease whose pathogenesis involves the interplay between venous stasis, hypercoagulability and endothelial damage. After more than a century since Virchow’s enumeration of these three elements, the concept of hypercoagulability and endothelial damage evolved substantially, and inflammation is currently recognized as a common cause of both alterations [51, 52]. According to this updated view, thrombo-inflammation, which involves leukocyte and platelet adhesion to the endothelium as well as local thrombin and fibrin generation, is part of a “biological response” that contributes to pathogen clearance and tissue repair, but that in the presence of prothrombotic factors (e.g. cancer, estrogens, etc) can tip the system towards a hypercoagulable state thereby triggering the cellular events of VTE [53–55]. On the other hand, the pathogenesis of CVD is intimately associated with atherogenesis, which involves (i) the recruitment, adherence and transmigration of circulating leukocytes to areas of endothelial damage; (ii) maturation of monocytes mature into macrophages in the intima which engulf low density lipoprotein molecules (originating foam cells); (iii) migration and proliferation of smooth muscle cells from the media into the intima, coupled with the synthesis of extracellular matrix molecules which contribute to the formation of the fibrous cap; and (iv) repeated cycles of proliferation and cell death inside the plaque which contribute to its growth and instability, and eventually lead to its physical rupture, which activates hemostasis (by tissue factor exposure and platelet activation), and ultimately results in thrombosis and ischemia [49, 52, 56].

Yet, several lines of evidence support an at least a partial overlap in the pathogenesis of VTE and CVD. From an epidemiological standpoint, this is well illustrated by a study that revealed that patients with unprovoked VTE present an estimated risk of atherosclerosis that is 5.1 and 14.5-fold higher than in patients with secondary VTE and healthy controls, respectively [7, 57] and by the existence of conditions that increase the risk of both VTE and CVD such as APS [58] and SCD [9, 59]. In addition, the cross-talk between the immune system, hemostasis and atherogenesis is being increasingly supported by experimental data [14, 15, 52, 60]. And finally, the classical borders between CVD and VTE were further blurred by results from large-scale clinical trials in which aspirin was shown to decrease the risk of recurrent VTE [10, 11], and rivaroxaban, an anti-factor Xa anticoagulant was shown to decrease the risk of recurrent CVD more effectively than aspirin [12].

Using an integrative bioinformatics approach we analyzed five independent datasets of gene expression data and generated two distinct lists of genes that are commonly (n = 472) or divergently (n = 124) expressed in VTE and CVD. We took advantage of publicly available datasets from five well-designed studies addressing other scientific questions, but that generated high-quality data, all using the same microarray platform applied to both patients and healthy controls, and with sufficient meta-data to allow inter-study comparisons. We also took advantage of new standardized bioinformatics methods to perform data processing, meta-analyses and functional analyses [23, 61, 62].

Among commonly DE genes we observed a predominance of genes associated with innate immunity. These included genes that have been previously associated with CVD in human studies such as *PTX3* (pentraxin 3) [63–65] and *S100A12* (EN-RAGE) [66, 67], as well as genes that have been associated with CVD only in animal studies such as *ANXA3* and *SLPI*, both shown to be up-regulated in rodent models of ischemic stroke [68, 69]. Our meta-analysis also identified a commonly down-regulated gene, *ID3* (Inhibitor of DNA Binding 3. HLH Protein), which is atheroprotective in animal models [70] and whose functional polymorphisms have been associated with atherosclerosis protection in several populational studies [70, 71]. In regard to VTE, *TBX21* (T-box 21), which was commonly down-regulated in our study, has been recently associated with the resolution of VTE in an animal model [72]. Though less frequent, genes that are more directly associated with hemostasis were also identified such as *SERPINB2* (plasminogen activator inhibitor 2), whose polymorphisms have been associated with recurrent CVD [73]. Finally, The identification of *BACH1 and BACH2*, which are involved in heme metabolism, is also of interest since we and others have shown that heme is a potential activator of hemostasis [74–76]. The pattern observed in the analysis of individual DE genes was confirmed by the gene set analysis that identified pathways associated with hemostasis and innate immunity as the most consistently associated with the gene signature of VTE and CVD. Of note, pathways whose associations with the pathogenesis, diagnosis and even treatment of VTE/CVD were only recently confirmed, such as IL-1 signaling and neutrophil mediated immunity, emerged with strong associations in our model.

Genes whose expression was discordant between VTE and CVD were also explored, and in addition to a full list of these genes, we opted to identify pathways that were over-represented in VTE compared to CVD and vice versa. The most significant result was the identification of several neutrophil-related pathways in CVD when compared to VTE, suggesting a more prominent role for these cells in the former. Some of the genes involved in these pathways have been associated with CVD in animal models (*MCL1*, *JUND*, *PELI1*) [77–79], and in humans (*ACSL1*, *AOC3*, *ALPL*, *MMP9*, *PPIF*, *GRK2*) [80–87]. Also of interest was the identification of *PADI4*, a critical enzyme for the formation of neutrophil extracellular traps [88], which has been previously associated with other vascular-related phenotypes in animal models [89, 90].

These results are of interest for the following reasons: first, they represent a confirmation that the participation of innate immunity and hemostasis in the pathophysiology of VTE and CVD is also evident at the transcriptomic level, an observation that to our knowledge had not been previously demonstrated at a systems biology level; and second, the list of genes and pathways identified in our study (provided in detailed supplementary lists) may allow other groups to gain new insights about the pathophysiology of VTE and CVD at the cellular level and molecular level, as well as the identification of new biomarkers or therapeutic targets. In favor of this possibility is unsupervised call of the IL-1 pathway as a relevant pathway in the pathogenesis of CVD, which was recently confirmed by the CANTOS clinical trial [91], as well as the identification of complement, intrinsic prothrombin activation and neutrophil function as enriched pathways in both VTE and CVD, which is in accordance with new and evolving concepts of hemostasis and thrombosis [16, 92–94]. We also validated our results using a robust machine learning strategy in independent cohorts, by demonstrating that a list of 107 divergently expressed genes derived from our analysis was capable to discriminate with 100% accuracy patients with VTE and AMI. In addition, three other gene classifiers were constructed to discriminate patients with VTE, AMI and IS from healthy individuals with a precisions of 76.5%, 91.6% and 81.4% respectively. While it should be emphasized that the objective of these validations is not to claim that these genes should be used to discriminate two conditions that are clearly defined by clinical characteristics, it does confirm that the experimental strategies used in our analyses are valid.

Our study has limitations that need to be acknowledged. As in any meta-analysis, results are dependent and limited by characteristics of the original studies. Even though we restricted our analysis to datasets used in peer-reviewed published studies, with high-quality meta-data and from the same microarray platform, only two studies involving VTE were available [40, 43], which were used as training and validation cohort. Since in both studies patients with cancer and APS were excluded, we believe that they although limited in number, they provide a good representation of VTE patients. The relative scarcity of microarray datasets was also the reason why we had to include studies using RNA from different sources (whole blood and PBMC), and with different time of sample collection since the index thrombotic event. In fact, this compromise between sample homogeneity and sample availability was necessary, or the study would not have been possible. Accordingly, we acknowledge that it is not possible to exclude that additional commonly expressed genes could have been identified if all datasets were from the same RNA source (type II error). On the other hand, since positive findings from our analytical approach were those that were remained significant in all samples despite this relative heterogeneity, our conclusions are likely to be of biological relevance (lower chance of type I error), as supported by our external validation. In fact, the concept of gene expression meta-analysis has been previously used in the context of other complex diseases [38, 95–97].

In conclusion, we demonstrate that the participation of innate immunity, complement and hemostasis activation in the pathogenesis of VTE and CVD is also evident at the transcriptomic level. We also demonstrate that in CVD, pathways associated with IL-1 signaling and neutrophil activation are relatively more represented in CVD than in VTE and that the gene expression signature of VTE resembles more closely the pattern observed in cardioembolic stroke than the pattern observed in AMI, IS or PAOD. Finally, we provide two validated lists of genes whose expression is shared or discordant between VTE and CVD, which can be used in future studies involving these two conditions.

## Supporting information

S1 Table List of all genes identified in the meta-analysis between VTE and CVD. (DOCX)

S2 Table Top differentially expressed genes identified in the meta-analysis of studies involving acute CVD. (DOCX)

S3 Table Top differentially expressed genes identified in the meta-analysis of studies involving chronic CVD. (DOCX)

S4 Table Divergently expressed genes in CVD and VTE. (DOCX)

S5 Table Genes that are up-regulated in CVD (vs VTE) expressed in neutrophils. (XLSX)

S1 Fig Correlation of gene expression changes studies of CVD whose samples were collected in the acute (a) or chronic (b) phase of their disease courses. Ischemic stroke (IS), Peripheral arterial occlusive disease (PAOD), Acute myocardial infarction (AMI) and Cardioembolic stroke (CS). Pairwise correlation scatter plots are in the lower triangle boxes. The upper triangle boxes show Pearson correlation coefficients (R) of log2 fold changes for all 472 differentially expressed genes identified in the meta-analysis of all 5 studies.
(DOCX)
