# Supplementary material for: Identification of common and divergent gene expression signatures in patients with venous and arterial thrombosis using data from public repositories
Source: PLoS One. 2020 Aug 11;15(8):e0235501. doi: 10.1371/journal.pone.0235501 (PMC7418995; doi:10.1371/journal.pone.0235501)
Supplement: S1 Table — (DOCX) [file pone.0235501.s001.docx]

**Supplementary table 1.** List of all genes identified in the meta-analysis between VTE and CVD

| Fold-change in individual studies (LogFC) | | | | | | Meta-analysis results | |
| --- | --- | --- | --- | --- | --- | --- | --- |
| Genes | VTE | PAOD | AMI | CS | IS | AveLogFC | FDR |
| Up-regulated genes | | | | | | | |
| *G0S2* | 0.26 | 1.96 | 0.16 | 0.82 | 2.29 | 2.14 | 0 |
| *BCL2A1* | 1.19 | 0.79 | 0.38 | 0.91 | 0.63 | 1.72 | 0 |
| *TNFAIP6* | 1.03 | 0.78 | 0.30 | 0.77 | 0.53 | 1.61 | 0 |
| *ANXA3* | 0.78 | 0.27 | 0.87 | 0.97 | 0.49 | 1.60 | 0 |
| *SERPINB2* | 0.25 | 0.50 | 0.30 | 0.97 | 0.93 | 1.50 | 0 |
| *S100A12* | 1.07 | 0.01 | 0.80 | 0.84 | 0.00 | 1.46 | 0 |
| *SLPI* | 0.88 | 0.00 | 0.67 | 0.72 | 0.19 | 1.41 | 0 |
| *FKBP1B* | 1.04 | 0.29 | 0.12 | 0.68 | 0.25 | 1.39 | 0 |
| *DEFA4* | 0.33 | 0.35 | 0.02 | 1.06 | 0.63 | 1.39 | 0 |
| *PTX3* | 0.08 | 0.64 | 0.16 | 0.44 | 0.99 | 1.38 | 2.17E-07 |
| *SAMSN1* | 0.53 | 0.51 | 0.11 | 0.55 | 0.55 | 1.37 | 0 |
| *S100P* | 0.48 | 0.35 | 0.44 | 0.72 | 0.25 | 1.37 | 0 |
| *ARG1* | 0.73 | 0.05 | 0.24 | 1.08 | 0.13 | 1.36 | 0 |
| *BPGM* | 1.26 | 0.26 | 0.15 | 0.09 | 0.44 | 1.36 | 0 |
| *EGR2* | 0.00 | 0.91 | 0.20 | 0.13 | 0.97 | 1.36 | 0.0003627 |
| *RNASE2* | 0.91 | 0.30 | 0.40 | 0.57 | 0.03 | 1.36 | 0 |
| *LPAR6* | 1.49 | 0.16 | 0.04 | 0.35 | 0.01 | 1.33 | 0 |
| *SLC22A4* | 0.12 | 0.44 | 0.40 | 0.77 | 0.27 | 1.32 | 0 |
| *EREG* | 0.01 | 0.76 | 0.03 | 0.06 | 1.13 | 1.32 | 0.02459 |
| *GYPB* | 0.92 | 0.17 | 0.16 | 0.47 | 0.28 | 1.32 | 0 |
| *IL1R2* | 0.13 | 0.34 | 0.47 | 0.68 | 0.34 | 1.31 | 0 |
| *FECH* | 1.03 | 0.08 | 0.03 | 0.52 | 0.20 | 1.30 | 0 |
| *CAMP* | 0.53 | 0.27 | 0.36 | 0.48 | 0.22 | 1.30 | 0 |
| *HBD* | 1.18 | 0.01 | 0.03 | 0.02 | 0.62 | 1.30 | 0 |
| *VSIG4* | 0.25 | 0.14 | 0.02 | 1.33 | 0.08 | 1.29 | 0 |
| *CEACAM6* | 0.04 | 0.29 | 0.08 | 1.04 | 0.36 | 1.29 | 0 |
| *CLEC4E* | 0.17 | 0.38 | 0.14 | 0.88 | 0.22 | 1.28 | 0 |
| *VNN1* | 0.17 | 0.51 | 0.46 | 0.53 | 0.12 | 1.28 | 0 |
| *CA1* | 0.77 | 0.06 | 0.11 | 0.39 | 0.45 | 1.28 | 0 |
| *OLAH* | 0.09 | 0.05 | 0.16 | 1.44 | 0.01 | 1.28 | 0.0002393 |
| *FHL2* | 0.53 | 0.12 | 0.11 | 0.66 | 0.23 | 1.26 | 0 |
| *RGS1* | 0.06 | 0.46 | 0.04 | 0.19 | 0.89 | 1.26 | 0.006703 |
| *CLIC2* | 0.81 | 0.06 | 0.04 | 0.66 | 0.05 | 1.25 | 0 |
| *STK17B* | 0.40 | 0.35 | 0.06 | 0.61 | 0.18 | 1.25 | 0 |
| *MMP8* | 0.21 | 0.29 | 0.20 | 0.56 | 0.34 | 1.25 | 0 |
| *PLK2* | 0.03 | 0.61 | 0.04 | 0.55 | 0.36 | 1.25 | 0.005566 |
| *ELANE* | 0.09 | 0.43 | 0.16 | 0.48 | 0.43 | 1.25 | 0 |
| *SMIM27* | 0.35 | 0.08 | 0.05 | 1.03 | 0.08 | 1.25 | 0 |
| *AQP9* | 0.00 | 0.57 | 0.53 | 0.34 | 0.13 | 1.24 | 3.61E-07 |
| *CXCL2* | 0.06 | 0.25 | 0.07 | 0.16 | 1.02 | 1.24 | 0.01682 |
| *ABCG2* | 0.66 | 0.11 | 0.07 | 0.57 | 0.14 | 1.24 | 0 |
| *MCTP1* | 0.72 | 0.09 | 0.10 | 0.63 | 0.01 | 1.24 | 0 |
| *RNASE4* | 0.26 | 0.43 | 0.19 | 0.65 | 0.02 | 1.24 | 0 |
| *SIAH2* | 0.58 | 0.27 | 0.12 | 0.21 | 0.33 | 1.23 | 0 |
| *CDKN1A* | 0.05 | 0.69 | 0.02 | 0.12 | 0.62 | 1.23 | 2.24E-06 |
| *BTNL8* | 0.01 | 0.12 | 0.04 | 1.25 | 0.08 | 1.23 | 0 |
| *F5* | 0.23 | 0.14 | 0.35 | 0.75 | 0.02 | 1.23 | 0 |
| *CRISP3* | 0.37 | 0.28 | 0.27 | 0.20 | 0.34 | 1.22 | 0 |
| *GPR15* | 0.03 | 0.73 | 0.28 | 0.41 | 0.01 | 1.22 | 0.0002798 |
| *F8* | 0.38 | 0.23 | 0.12 | 0.61 | 0.12 | 1.22 | 0 |
| *SNX7* | 0.18 | 0.32 | 0.18 | 0.66 | 0.10 | 1.22 | 2.24E-06 |
| *MAOA* | 0.19 | 0.04 | 0.01 | 1.14 | 0.06 | 1.22 | 3.49E-05 |
| *MBOAT2* | 0.02 | 0.18 | 0.29 | 0.72 | 0.17 | 1.21 | 1.64E-05 |
| *BEX1* | 0.08 | 0.17 | 0.02 | 0.79 | 0.30 | 1.21 | 2.14E-05 |
| *MAFF* | 0.03 | 0.41 | 0.05 | 0.15 | 0.73 | 1.21 | 0.003426 |
| *ABCA1* | 0.32 | 0.08 | 0.14 | 0.71 | 0.09 | 1.20 | 0 |
| *CEACAM8* | 0.07 | 0.17 | 0.12 | 0.65 | 0.30 | 1.20 | 0 |
| *PHACTR1* | 0.55 | 0.04 | 0.05 | 0.34 | 0.29 | 1.19 | 0 |
| *CREG1* | 0.88 | 0.11 | 0.06 | 0.20 | 0.01 | 1.19 | 0 |
| *CLEC1A* | 0.03 | 0.05 | 0.19 | 0.91 | 0.09 | 1.19 | 4.68E-05 |
| *RCAN2* | 0.08 | 0.16 | 0.00 | 0.89 | 0.11 | 1.19 | 0.0003004 |
| *GRB10* | 0.18 | 0.04 | 0.17 | 0.83 | 0.02 | 1.19 | 5.77E-07 |
| *IRAK3* | 0.04 | 0.12 | 0.26 | 0.80 | 0.01 | 1.19 | 0 |
| *LTF* | 0.02 | 0.07 | 0.25 | 0.45 | 0.44 | 1.19 | 0 |
| *SAP30* | 0.17 | 0.21 | 0.05 | 0.70 | 0.06 | 1.18 | 0.0005664 |
| *RNASE3* | 0.12 | 0.17 | 0.07 | 0.68 | 0.16 | 1.18 | 0 |
| *CTSG* | 0.00 | 0.16 | 0.07 | 0.42 | 0.55 | 1.18 | 5.77E-07 |
| *HPSE* | 0.10 | 0.28 | 0.17 | 0.60 | 0.03 | 1.18 | 3.61E-07 |
| *C9orf78* | 0.66 | 0.08 | 0.08 | 0.19 | 0.14 | 1.17 | 0 |
| *QPCT* | 0.00 | 0.31 | 0.34 | 0.38 | 0.12 | 1.17 | 7.22E-08 |
| *ADORA2B* | 0.22 | 0.33 | 0.05 | 0.49 | 0.06 | 1.17 | 3.68E-06 |
| *HDGFL3* | 0.16 | 0.26 | 0.05 | 0.36 | 0.31 | 1.17 | 0.002335 |
| *MS4A3* | 0.33 | 0.30 | 0.03 | 0.22 | 0.25 | 1.17 | 0 |
| *SERPINB8* | 0.17 | 0.03 | 0.08 | 0.60 | 0.24 | 1.17 | 2.84E-05 |
| *SPATA2L* | 0.45 | 0.07 | 0.10 | 0.40 | 0.08 | 1.17 | 0 |
| *PDZD8* | 0.04 | 0.32 | 0.07 | 0.46 | 0.20 | 1.16 | 1.08E-05 |
| *ADAM9* | 0.39 | 0.13 | 0.06 | 0.50 | 0.02 | 1.16 | 0 |
| *MT1E* | 0.39 | 0.20 | 0.19 | 0.25 | 0.05 | 1.16 | 0 |
| *LY6G5C* | 0.02 | 0.16 | 0.03 | 0.65 | 0.21 | 1.16 | 0.000438 |
| *OSBPL1A* | 0.19 | 0.08 | 0.11 | 0.67 | 0.01 | 1.16 | 2.14E-05 |
| *CAVIN3* | 0.31 | 0.25 | 0.11 | 0.37 | 0.00 | 1.16 | 0 |
| *SLC7A5* | 0.17 | 0.12 | 0.05 | 0.38 | 0.33 | 1.16 | 0 |
| *KIF13A* | 0.41 | 0.07 | 0.26 | 0.29 | 0.02 | 1.16 | 0 |
| *CPQ* | 0.08 | 0.06 | 0.14 | 0.68 | 0.08 | 1.16 | 0.001604 |
| *PI3* | 0.07 | 0.21 | 0.53 | 0.17 | 0.06 | 1.16 | 0 |
| *NEIL3* | 0.06 | 0.07 | 0.08 | 0.69 | 0.12 | 1.15 | 0.0428 |
| *ORM1* | 0.20 | 0.09 | 0.23 | 0.23 | 0.25 | 1.15 | 0 |
| *NENF* | 0.11 | 0.04 | 0.06 | 0.73 | 0.06 | 1.15 | 0.006041 |
| *PLBD1* | 0.06 | 0.18 | 0.48 | 0.24 | 0.04 | 1.15 | 3.39E-06 |
| *FMO4* | 0.06 | 0.22 | 0.10 | 0.50 | 0.10 | 1.15 | 0.02705 |
| *IL1RAP* | 0.14 | 0.12 | 0.09 | 0.37 | 0.26 | 1.15 | 7.94E-07 |
| *CCRL2* | 0.29 | 0.16 | 0.04 | 0.38 | 0.12 | 1.15 | 7.22E-08 |
| *PAPSS1* | 0.23 | 0.10 | 0.17 | 0.46 | 0.02 | 1.14 | 2.17E-07 |
| *TFDP1* | 0.13 | 0.04 | 0.03 | 0.68 | 0.09 | 1.14 | 5.77E-07 |
| *FAS* | 0.25 | 0.38 | 0.08 | 0.05 | 0.20 | 1.14 | 0 |
| *NSUN3* | 0.48 | 0.19 | 0.02 | 0.22 | 0.05 | 1.14 | 0 |
| *OASL* | 0.16 | 0.56 | 0.05 | 0.09 | 0.08 | 1.14 | 0 |
| *FTH1* | 0.14 | 0.30 | 0.02 | 0.26 | 0.23 | 1.14 | 0.01135 |
| *HIST1H1T* | 0.46 | 0.00 | 0.03 | 0.39 | 0.07 | 1.14 | 0 |
| *NRG1* | 0.32 | 0.05 | 0.24 | 0.32 | 0.01 | 1.14 | 0 |
| *MPP1* | 0.19 | 0.29 | 0.20 | 0.02 | 0.23 | 1.14 | 0 |
| *NEFL* | 0.44 | 0.17 | 0.07 | 0.07 | 0.19 | 1.14 | 0 |
| *RP2* | 0.09 | 0.15 | 0.05 | 0.46 | 0.17 | 1.14 | 0 |
| *ATF6* | 0.29 | 0.17 | 0.10 | 0.34 | 0.01 | 1.14 | 2.17E-07 |
| *ASPH* | 0.07 | 0.03 | 0.19 | 0.55 | 0.05 | 1.13 | 0.02724 |
| *ATXN1* | 0.33 | 0.00 | 0.01 | 0.49 | 0.04 | 1.13 | 0 |
| *NXT1* | 0.20 | 0.16 | 0.05 | 0.28 | 0.19 | 1.13 | 0.0004492 |
| *ZDHHC3* | 0.40 | 0.09 | 0.08 | 0.30 | 0.01 | 1.13 | 0 |
| *MAP3K20* | 0.09 | 0.11 | 0.13 | 0.52 | 0.01 | 1.13 | 0.01782 |
| *ATP11B* | 0.24 | 0.11 | 0.02 | 0.43 | 0.05 | 1.13 | 2.17E-07 |
| *KLF5* | 0.19 | 0.26 | 0.09 | 0.06 | 0.26 | 1.13 | 3.27E-05 |
| *NRIP3* | 0.16 | 0.04 | 0.09 | 0.39 | 0.18 | 1.13 | 0.000233 |
| *ZEB1* | 0.15 | 0.06 | 0.06 | 0.46 | 0.11 | 1.12 | 0.0003027 |
| *TAF7* | 0.53 | 0.11 | 0.03 | 0.13 | 0.03 | 1.12 | 0 |
| *TIMM17B* | 0.35 | 0.11 | 0.08 | 0.17 | 0.12 | 1.12 | 2.17E-07 |
| *CTSE* | 0.28 | 0.02 | 0.30 | 0.09 | 0.14 | 1.12 | 0 |
| *RAP1GAP* | 0.45 | 0.06 | 0.03 | 0.21 | 0.07 | 1.12 | 0 |
| *SSFA2* | 0.20 | 0.38 | 0.01 | 0.19 | 0.04 | 1.12 | 3.61E-07 |
| *OLR1* | 0.10 | 0.03 | 0.05 | 0.45 | 0.18 | 1.12 | 0.0008387 |
| *ARG2* | 0.22 | 0.15 | 0.02 | 0.26 | 0.16 | 1.12 | 2.13E-05 |
| *RHD* | 0.38 | 0.09 | 0.04 | 0.21 | 0.08 | 1.12 | 0 |
| *ACPP* | 0.02 | 0.10 | 0.15 | 0.43 | 0.06 | 1.11 | 0.006282 |
| *CRAT* | 0.13 | 0.09 | 0.09 | 0.39 | 0.07 | 1.11 | 8.66E-06 |
| *HIST1H1C* | 0.14 | 0.32 | 0.07 | 0.21 | 0.04 | 1.11 | 3.61E-07 |
| *KAZN* | 0.02 | 0.10 | 0.10 | 0.46 | 0.09 | 1.11 | 0.0002883 |
| *PCOLCE2* | 0.09 | 0.04 | 0.10 | 0.45 | 0.09 | 1.11 | 0.01945 |
| *SMPDL3A* | 0.07 | 0.14 | 0.15 | 0.28 | 0.12 | 1.11 | 5.20E-06 |
| *DDIT3* | 0.09 | 0.16 | 0.19 | 0.16 | 0.16 | 1.11 | 0.01173 |
| *LTBP2* | 0.30 | 0.04 | 0.03 | 0.22 | 0.16 | 1.11 | 0 |
| *CDC42* | 0.23 | 0.09 | 0.03 | 0.27 | 0.12 | 1.11 | 4.19E-05 |
| *PQLC1* | 0.40 | 0.04 | 0.11 | 0.16 | 0.03 | 1.11 | 0 |
| *MYL6* | 0.49 | 0.05 | 0.01 | 0.17 | 0.00 | 1.11 | 0 |
| *ELL2* | 0.27 | 0.06 | 0.04 | 0.29 | 0.06 | 1.11 | 4.04E-06 |
| *PFDN1* | 0.26 | 0.12 | 0.02 | 0.29 | 0.02 | 1.11 | 0.0004352 |
| *PLOD2* | 0.11 | 0.43 | 0.01 | 0.02 | 0.16 | 1.11 | 0.01309 |
| *STX8* | 0.58 | 0.07 | 0.00 | 0.05 | 0.03 | 1.11 | 0 |
| *CETP* | 0.06 | 0.14 | 0.14 | 0.31 | 0.07 | 1.10 | 0.0005939 |
| *NDUFA7* | 0.58 | 0.04 | 0.02 | 0.04 | 0.02 | 1.10 | 0 |
| *HIST1H2AJ* | 0.08 | 0.35 | 0.01 | 0.12 | 0.15 | 1.10 | 0.03903 |
| *PIP5K1B* | 0.17 | 0.03 | 0.06 | 0.38 | 0.07 | 1.10 | 0.0004065 |
| *TNFSF8* | 0.24 | 0.04 | 0.07 | 0.17 | 0.17 | 1.10 | 1.30E-06 |
| *ANAPC15* | 0.29 | 0.12 | 0.14 | 0.07 | 0.07 | 1.10 | 3.90E-05 |
| *TMX4* | 0.12 | 0.18 | 0.00 | 0.27 | 0.11 | 1.10 | 0.01225 |
| *PSMD9* | 0.20 | 0.00 | 0.10 | 0.35 | 0.01 | 1.10 | 0.004433 |
| *MGST3* | 0.40 | 0.04 | 0.03 | 0.14 | 0.05 | 1.10 | 2.17E-07 |
| *KRAS* | 0.31 | 0.06 | 0.03 | 0.13 | 0.11 | 1.09 | 1.17E-05 |
| *SV2A* | 0.05 | 0.02 | 0.09 | 0.26 | 0.21 | 1.09 | 0.01888 |
| *FOXO3* | 0.21 | 0.06 | 0.00 | 0.21 | 0.14 | 1.09 | 2.17E-07 |
| *LRPAP1* | 0.16 | 0.00 | 0.01 | 0.44 | 0.01 | 1.09 | 0.02262 |
| *ZNF668* | 0.09 | 0.05 | 0.09 | 0.37 | 0.02 | 1.09 | 0.03108 |
| *UBE2D1* | 0.01 | 0.11 | 0.14 | 0.27 | 0.07 | 1.09 | 0.003432 |
| *AP3S1* | 0.21 | 0.11 | 0.04 | 0.23 | 0.00 | 1.09 | 0.0002054 |
| *C3AR1* | 0.08 | 0.08 | 0.11 | 0.33 | 0.00 | 1.09 | 4.94E-05 |
| *KANK2* | 0.25 | 0.07 | 0.13 | 0.06 | 0.08 | 1.09 | 1.44E-07 |
| *MARCH2* | 0.03 | 0.21 | 0.02 | 0.14 | 0.18 | 1.08 | 5.77E-07 |
| *ZFAND5* | 0.06 | 0.15 | 0.02 | 0.16 | 0.19 | 1.08 | 0.02854 |
| *AVPI1* | 0.07 | 0.05 | 0.02 | 0.30 | 0.14 | 1.08 | 0.003737 |
| *CXCL9* | 0.07 | 0.21 | 0.03 | 0.26 | 0.00 | 1.08 | 0.01673 |
| *CCR8* | 0.06 | 0.15 | 0.13 | 0.08 | 0.13 | 1.08 | 0.02369 |
| *KLF7* | 0.05 | 0.11 | 0.09 | 0.28 | 0.02 | 1.08 | 0.003742 |
| *CHRNA10* | 0.11 | 0.05 | 0.02 | 0.33 | 0.03 | 1.08 | 0.02015 |
| *GREM2* | 0.16 | 0.17 | 0.06 | 0.11 | 0.05 | 1.08 | 0.0101 |
| *SLC6A9* | 0.08 | 0.12 | 0.13 | 0.17 | 0.05 | 1.08 | 5.88E-05 |
| *ARHGEF12* | 0.28 | 0.07 | 0.02 | 0.12 | 0.07 | 1.08 | 4.83E-06 |
| *HIST1H2BK* | 0.05 | 0.12 | 0.01 | 0.21 | 0.17 | 1.08 | 0.002055 |
| *OAT* | 0.23 | 0.14 | 0.01 | 0.07 | 0.10 | 1.08 | 1.03E-05 |
| *NME4* | 0.36 | 0.01 | 0.01 | 0.16 | 0.01 | 1.08 | 0 |
| *HBB* | 0.29 | 0.15 | 0.00 | 0.08 | 0.02 | 1.08 | 8.66E-07 |
| *ST3GAL6* | 0.13 | 0.01 | 0.15 | 0.20 | 0.04 | 1.08 | 0.0001827 |
| *MYLIP* | 0.13 | 0.03 | 0.02 | 0.20 | 0.15 | 1.08 | 0.001208 |
| *NINJ2* | 0.24 | 0.06 | 0.04 | 0.14 | 0.04 | 1.07 | 0 |
| *GABRR2* | 0.10 | 0.01 | 0.20 | 0.06 | 0.13 | 1.07 | 0.01089 |
| *LAPTM4A* | 0.25 | 0.12 | 0.03 | 0.09 | 0.01 | 1.07 | 0.000385 |
| *LDHA* | 0.19 | 0.03 | 0.11 | 0.15 | 0.02 | 1.07 | 0.003539 |
| *DERL2* | 0.43 | 0.00 | 0.01 | 0.04 | 0.00 | 1.07 | 7.94E-07 |
| *CD33* | 0.14 | 0.03 | 0.10 | 0.18 | 0.05 | 1.07 | 0.001163 |
| *GRB7* | 0.06 | 0.14 | 0.16 | 0.06 | 0.07 | 1.07 | 0.04529 |
| *KCNE2* | 0.08 | 0.05 | 0.09 | 0.18 | 0.08 | 1.07 | 0.01886 |
| *TWF1* | 0.30 | 0.05 | 0.02 | 0.07 | 0.04 | 1.07 | 0.0002189 |
| *GDF5* | 0.03 | 0.05 | 0.11 | 0.06 | 0.21 | 1.07 | 0.04412 |
| *SUSD4* | 0.02 | 0.13 | 0.09 | 0.06 | 0.17 | 1.07 | 0.01066 |
| *BTD* | 0.20 | 0.07 | 0.02 | 0.18 | 0.00 | 1.07 | 0.01935 |
| *WIPI1* | 0.03 | 0.12 | 0.10 | 0.17 | 0.05 | 1.07 | 0.02413 |
| *HIST1H2AM* | 0.10 | 0.10 | 0.04 | 0.02 | 0.19 | 1.07 | 0.001708 |
| *TFRC* | 0.23 | 0.02 | 0.01 | 0.09 | 0.10 | 1.06 | 6.77E-05 |
| *CRTAP* | 0.18 | 0.01 | 0.03 | 0.19 | 0.03 | 1.06 | 0.0195 |
| *GOLM1* | 0.02 | 0.13 | 0.05 | 0.22 | 0.01 | 1.06 | 0.04014 |
| *TCF15* | 0.02 | 0.10 | 0.05 | 0.15 | 0.10 | 1.06 | 0.03079 |
| *CENPX* | 0.24 | 0.00 | 0.08 | 0.09 | 0.00 | 1.06 | 0.001626 |
| *NPM3* | 0.13 | 0.09 | 0.02 | 0.05 | 0.12 | 1.06 | 0.04017 |
| *LRFN3* | 0.07 | 0.01 | 0.02 | 0.21 | 0.09 | 1.06 | 0.004148 |
| *RAB25* | 0.10 | 0.05 | 0.13 | 0.08 | 0.03 | 1.06 | 0.0283 |
| *KRT3* | 0.11 | 0.04 | 0.05 | 0.02 | 0.17 | 1.06 | 0.03276 |
| *BCAM* | 0.04 | 0.12 | 0.08 | 0.05 | 0.09 | 1.05 | 0.0454 |
| *DSE* | 0.17 | 0.04 | 0.05 | 0.10 | 0.02 | 1.05 | 4.69E-06 |
| *ARID5B* | 0.12 | 0.02 | 0.03 | 0.09 | 0.12 | 1.05 | 0.001748 |
| *SMOX* | 0.01 | 0.18 | 0.01 | 0.00 | 0.15 | 1.05 | 0.0003396 |
| *CPA4* | 0.06 | 0.09 | 0.04 | 0.02 | 0.14 | 1.05 | 0.02986 |
| *HERC6* | 0.18 | 0.02 | 0.08 | 0.04 | 0.01 | 1.05 | 0.001743 |
| *ALLC* | 0.14 | 0.01 | 0.02 | 0.09 | 0.08 | 1.05 | 0.01064 |
| *CD8B* | 0.08 | 0.04 | 0.10 | 0.06 | 0.06 | 1.05 | 7.94E-07 |
| *MITF* | 0.16 | 0.06 | 0.05 | 0.05 | 0.01 | 1.05 | 0.002087 |
| *PRSS50* | 0.10 | 0.06 | 0.10 | 0.05 | 0.00 | 1.05 | 0.02529 |
| *NUBP2* | 0.17 | 0.04 | 0.03 | 0.06 | 0.00 | 1.04 | 0.02948 |
| *RIT1* | 0.10 | 0.06 | 0.04 | 0.05 | 0.04 | 1.04 | 0.0002302 |
| *KLHDC8A* | 0.12 | 0.05 | 0.02 | 0.05 | 0.04 | 1.04 | 0.002746 |
| *USP13* | 0.12 | 0.05 | 0.04 | 0.01 | 0.05 | 1.04 | 0.02251 |
| *CYP26B1* | 0.08 | 0.01 | 0.03 | 0.06 | 0.07 | 1.04 | 0.03082 |
| Down-regulated genes | | | | | | | |
| *CLIC3* | -0.14 | -0.08 | -0.49 | -1.07 | -0.19 | 0.76 | 0 |
| *BACH2* | -0.31 | -0.46 | -0.18 | -0.71 | -0.08 | 0.79 | 0 |
| *TXK* | -0.24 | -0.36 | -0.44 | -0.43 | -0.24 | 0.79 | 0 |
| *MLC1* | -0.21 | -0.16 | -0.27 | -0.86 | -0.19 | 0.79 | 0 |
| *ID3* | -0.10 | -0.35 | -0.15 | -0.82 | -0.24 | 0.79 | 0 |
| *ZNF304* | -0.14 | -0.29 | -0.36 | -0.25 | -0.58 | 0.80 | 0 |
| *EVL* | -0.35 | -0.32 | -0.22 | -0.55 | -0.13 | 0.81 | 0 |
| *BCOR* | -0.57 | -0.03 | -0.30 | -0.52 | -0.07 | 0.81 | 0 |
| *TBX21* | -0.27 | -0.24 | -0.55 | -0.13 | -0.27 | 0.82 | 0 |
| *IL2RB* | -0.23 | -0.20 | -0.50 | -0.45 | -0.06 | 0.82 | 0 |
| *TRIB2* | -0.47 | -0.07 | -0.26 | -0.53 | -0.11 | 0.82 | 0 |
| *CCR7* | -0.06 | -0.39 | -0.13 | -0.76 | -0.07 | 0.82 | 0 |
| *PRF1* | -0.24 | -0.22 | -0.56 | -0.15 | -0.21 | 0.83 | 0 |
| *COL5A3* | -0.24 | -0.03 | 0.00 | -0.99 | -0.04 | 0.83 | 0 |
| *LUC7L* | -0.24 | -0.11 | -0.19 | -0.67 | -0.08 | 0.83 | 0 |
| *CCNL2* | -0.52 | -0.19 | -0.09 | -0.41 | -0.06 | 0.84 | 1.44E-07 |
| *HERC2* | -0.47 | -0.24 | -0.12 | -0.41 | -0.02 | 0.84 | 0 |
| *FCMR* | -0.34 | -0.29 | -0.11 | -0.43 | -0.10 | 0.84 | 0 |
| *TMC6* | -0.24 | -0.03 | -0.16 | -0.77 | -0.03 | 0.84 | 0 |
| *FMNL1* | -0.85 | -0.13 | -0.09 | -0.08 | -0.08 | 0.84 | 2.17E-07 |
| *LBH* | -0.47 | -0.27 | -0.23 | -0.12 | -0.13 | 0.84 | 0 |
| *CD247* | -0.09 | -0.15 | -0.41 | -0.50 | -0.06 | 0.85 | 0 |
| *MTR* | -0.34 | -0.06 | -0.30 | -0.41 | -0.09 | 0.85 | 2.96E-05 |
| *ATP2A3* | -0.68 | -0.11 | -0.01 | -0.39 | -0.01 | 0.85 | 0 |
| *PPWD1* | -0.19 | -0.19 | -0.26 | -0.39 | -0.17 | 0.85 | 0 |
| *NUP210* | -0.17 | -0.23 | -0.06 | -0.64 | -0.09 | 0.85 | 0 |
| *ZHX2* | -0.28 | -0.34 | -0.10 | -0.39 | -0.07 | 0.85 | 0.000163 |
| *WDR11* | -0.19 | -0.35 | -0.35 | -0.03 | -0.26 | 0.85 | 1.44E-07 |
| *NUMA1* | -0.43 | -0.10 | -0.14 | -0.41 | -0.09 | 0.85 | 0 |
| *PCSK7* | -0.26 | -0.24 | -0.25 | -0.27 | -0.15 | 0.85 | 0 |
| *BRD1* | -0.51 | -0.03 | -0.15 | -0.38 | -0.08 | 0.85 | 1.44E-07 |
| *MTA1* | -0.48 | -0.14 | 0.00 | -0.37 | -0.16 | 0.85 | 0 |
| *TNFRSF25* | -0.26 | -0.05 | -0.10 | -0.73 | -0.01 | 0.85 | 0 |
| *SLC7A6* | -0.52 | -0.15 | -0.17 | -0.29 | -0.03 | 0.85 | 7.22E-08 |
| *OFD1* | -0.02 | -0.34 | -0.30 | -0.33 | -0.15 | 0.85 | 0 |
| *CX3CR1* | -0.14 | -0.29 | -0.32 | -0.08 | -0.32 | 0.85 | 0.000486 |
| *KHDC4* | -0.19 | -0.31 | -0.23 | -0.11 | -0.30 | 0.85 | 7.22E-07 |
| *C11orf80* | -0.09 | -0.34 | -0.12 | -0.49 | -0.10 | 0.85 | 9.96E-06 |
| *DENND2D* | -0.13 | -0.32 | -0.30 | -0.21 | -0.17 | 0.85 | 7.00E-05 |
| *APBA2* | -0.21 | -0.14 | -0.04 | -0.71 | -0.03 | 0.85 | 5.92E-05 |
| *ZNF37BP* | -0.17 | -0.28 | -0.24 | -0.28 | -0.15 | 0.86 | 1.44E-07 |
| *NSUN5P1* | -0.26 | -0.23 | -0.07 | -0.47 | -0.08 | 0.86 | 2.89E-07 |
| *DFFB* | -0.27 | -0.07 | -0.17 | -0.46 | -0.14 | 0.86 | 1.44E-07 |
| *FNBP4* | -0.35 | -0.18 | -0.26 | -0.21 | -0.09 | 0.86 | 0 |
| *METTL3* | -0.25 | -0.28 | -0.22 | -0.24 | -0.11 | 0.86 | 0 |
| *PSMD3* | -0.36 | -0.04 | -0.03 | -0.55 | -0.09 | 0.86 | 2.38E-06 |
| *SYMPK* | -0.32 | -0.06 | -0.09 | -0.49 | -0.11 | 0.86 | 0 |
| *SLC6A16* | -0.24 | -0.33 | -0.16 | -0.26 | -0.07 | 0.86 | 0 |
| *ADGRG1* | -0.31 | -0.12 | -0.25 | -0.35 | -0.03 | 0.86 | 0 |
| *C1orf109* | -0.11 | -0.22 | -0.30 | -0.35 | -0.09 | 0.86 | 0 |
| *SETD1B* | -0.50 | -0.18 | -0.05 | -0.26 | -0.06 | 0.86 | 0.000374 |
| *ITM2C* | -0.24 | -0.01 | 0.00 | -0.59 | -0.21 | 0.87 | 0 |
| *ZNF623* | -0.04 | -0.20 | -0.29 | -0.26 | -0.24 | 0.87 | 1.44E-07 |
| *TCAF1* | -0.19 | -0.06 | -0.18 | -0.51 | -0.09 | 0.87 | 0.002007 |
| *CLSTN1* | -0.23 | -0.09 | -0.08 | -0.60 | -0.03 | 0.87 | 0.000345 |
| *CD38* | -0.17 | -0.16 | -0.19 | -0.11 | -0.39 | 0.87 | 5.77E-07 |
| *PFAS* | -0.51 | -0.19 | -0.11 | -0.18 | -0.04 | 0.87 | 0 |
| *RNF44* | -0.69 | -0.07 | -0.01 | -0.24 | -0.01 | 0.87 | 0 |
| *CXCR6* | -0.16 | -0.27 | -0.08 | -0.22 | -0.28 | 0.87 | 0 |
| *SAFB* | -0.43 | -0.02 | -0.04 | -0.39 | -0.13 | 0.87 | 1.44E-07 |
| *BTBD18* | -0.30 | -0.30 | -0.24 | 0.00 | -0.17 | 0.87 | 0 |
| *MDN1* | -0.39 | -0.21 | -0.11 | -0.24 | -0.05 | 0.87 | 0 |
| *PEX12* | -0.11 | -0.16 | -0.16 | -0.25 | -0.32 | 0.87 | 0 |
| *XRCC2* | 0.00 | -0.05 | -0.06 | -0.76 | -0.12 | 0.87 | 0.000314 |
| *SCRN1* | -0.04 | -0.05 | -0.20 | -0.42 | -0.29 | 0.87 | 0.000432 |
| *ANKFY1* | -0.57 | -0.04 | -0.06 | -0.21 | -0.10 | 0.87 | 0.000231 |
| *GANAB* | -0.54 | -0.02 | -0.07 | -0.23 | -0.13 | 0.87 | 0 |
| *GZMM* | -0.21 | -0.13 | -0.16 | -0.41 | -0.06 | 0.87 | 0 |
| *ZNF721* | -0.03 | -0.25 | -0.12 | -0.48 | -0.10 | 0.87 | 0 |
| *MCM7* | -0.21 | -0.18 | -0.05 | -0.44 | -0.10 | 0.87 | 0.005125 |
| *PTCD3* | 0.00 | -0.28 | -0.22 | -0.27 | -0.20 | 0.87 | 0.000279 |
| *EDEM1* | -0.26 | -0.05 | -0.18 | -0.47 | -0.01 | 0.87 | 0.04035 |
| *PUS7* | -0.33 | -0.02 | -0.08 | -0.30 | -0.24 | 0.87 | 3.18E-06 |
| *ARHGEF18* | -0.45 | -0.19 | -0.10 | -0.22 | 0.00 | 0.87 | 0 |
| *NONO* | -0.48 | -0.09 | -0.06 | -0.16 | -0.18 | 0.87 | 0 |
| *CIRBP* | -0.35 | -0.19 | -0.06 | -0.24 | -0.12 | 0.88 | 0 |
| *ABL1* | -0.39 | -0.06 | -0.03 | -0.44 | -0.03 | 0.88 | 0 |
| *ADAMTS1* | -0.06 | -0.14 | -0.29 | -0.30 | -0.15 | 0.88 | 0 |
| *SYNRG* | -0.27 | -0.10 | -0.15 | -0.23 | -0.19 | 0.88 | 0.000119 |
| *DROSHA* | -0.24 | -0.18 | -0.14 | -0.32 | -0.07 | 0.88 | 6.13E-06 |
| *CTCF* | -0.37 | -0.04 | -0.08 | -0.37 | -0.09 | 0.88 | 4.33E-06 |
| *HOXB2* | -0.27 | -0.01 | -0.27 | -0.15 | -0.24 | 0.88 | 0 |
| *RBM14* | -0.37 | -0.08 | -0.09 | -0.18 | -0.23 | 0.88 | 0 |
| *NOC2L* | -0.26 | -0.02 | -0.10 | -0.30 | -0.27 | 0.88 | 0 |
| *CBFA2T2* | -0.28 | -0.14 | -0.11 | -0.30 | -0.11 | 0.88 | 3.25E-06 |
| *PAN2* | -0.39 | -0.11 | -0.06 | -0.35 | -0.03 | 0.88 | 6.11E-05 |
| *LRCH3* | -0.14 | -0.22 | -0.23 | -0.20 | -0.14 | 0.88 | 0 |
| *YLPM1* | -0.49 | -0.08 | -0.14 | -0.18 | -0.03 | 0.88 | 0.000182 |
| *SEPT9* | -0.19 | -0.08 | -0.11 | -0.45 | -0.09 | 0.88 | 0 |
| *SFSWAP* | -0.18 | 0.00 | -0.14 | -0.52 | -0.09 | 0.88 | 0.000304 |
| *EML4* | -0.60 | -0.14 | -0.12 | -0.03 | -0.02 | 0.88 | 0.000491 |
| *ESYT1* | -0.32 | -0.11 | -0.05 | -0.39 | -0.04 | 0.88 | 0 |
| *ANKZF1* | -0.23 | -0.14 | -0.05 | -0.43 | -0.07 | 0.88 | 0 |
| *SLC20A1* | -0.19 | -0.22 | -0.19 | -0.13 | -0.18 | 0.88 | 2.71E-05 |
| *UTRN* | -0.57 | -0.01 | -0.16 | -0.01 | -0.17 | 0.88 | 8.08E-05 |
| *NSUN5* | -0.18 | -0.22 | -0.01 | -0.43 | -0.07 | 0.88 | 0 |
| *NMT2* | -0.05 | -0.25 | -0.23 | -0.30 | -0.08 | 0.88 | 0.000425 |
| *TUBB* | -0.20 | -0.01 | -0.08 | -0.45 | -0.16 | 0.88 | 6.57E-05 |
| *SATB1* | -0.01 | -0.48 | -0.24 | -0.04 | -0.13 | 0.88 | 0.00101 |
| *SPECC1L* | -0.24 | -0.14 | -0.08 | -0.41 | -0.04 | 0.88 | 0.01089 |
| *GTF3C1* | -0.33 | -0.12 | -0.06 | -0.36 | -0.01 | 0.88 | 5.20E-05 |
| *DNMT1* | -0.35 | 0.00 | -0.18 | -0.34 | -0.01 | 0.88 | 0 |
| *IKBKB* | -0.32 | -0.06 | -0.14 | -0.22 | -0.14 | 0.89 | 0 |
| *MORC2* | -0.17 | -0.19 | -0.09 | -0.29 | -0.13 | 0.89 | 1.01E-06 |
| *CCND3* | -0.16 | -0.19 | -0.07 | -0.38 | -0.07 | 0.89 | 0.000183 |
| *GCN1* | -0.22 | -0.21 | -0.03 | -0.31 | -0.10 | 0.89 | 0.007702 |
| *RABGGTB* | -0.08 | 0.00 | -0.21 | -0.52 | -0.06 | 0.89 | 0.000149 |
| *TAF15* | -0.27 | -0.13 | -0.26 | -0.10 | -0.11 | 0.89 | 0.000123 |
| *CARMIL1* | -0.12 | -0.20 | -0.05 | -0.43 | -0.06 | 0.89 | 1.44E-07 |
| *DOCK10* | -0.07 | -0.11 | -0.22 | -0.35 | -0.12 | 0.89 | 0.01847 |
| *S1PR5* | -0.31 | -0.09 | -0.22 | -0.23 | -0.01 | 0.89 | 0.00407 |
| *PIP5K1A* | -0.55 | -0.01 | -0.01 | -0.27 | -0.01 | 0.89 | 0 |
| *SEMA4C* | -0.30 | -0.07 | -0.06 | -0.42 | -0.01 | 0.89 | 0 |
| *EP400* | -0.45 | -0.02 | 0.00 | -0.32 | -0.05 | 0.89 | 1.95E-06 |
| *FEZ1* | -0.05 | -0.17 | -0.25 | -0.34 | -0.03 | 0.89 | 0 |
| *TARBP1* | -0.29 | -0.19 | -0.19 | -0.12 | -0.06 | 0.89 | 0.001935 |
| *ZNF45* | -0.08 | -0.07 | -0.14 | -0.47 | -0.09 | 0.89 | 2.17E-07 |
| *GTF2I* | -0.27 | -0.03 | -0.11 | -0.21 | -0.21 | 0.89 | 0.000113 |
| *ABCB1* | -0.09 | -0.07 | -0.33 | -0.31 | -0.03 | 0.89 | 1.95E-06 |
| *IL21R* | -0.11 | -0.17 | -0.09 | -0.36 | -0.11 | 0.89 | 6.03E-05 |
| *PRPF8* | -0.57 | -0.13 | -0.05 | -0.03 | -0.06 | 0.89 | 0.000846 |
| *CNTRL* | -0.43 | -0.07 | -0.17 | -0.01 | -0.14 | 0.89 | 0 |
| *SFI1* | -0.22 | -0.15 | -0.01 | -0.38 | -0.06 | 0.89 | 0 |
| *CEP164* | -0.27 | -0.03 | -0.01 | -0.41 | -0.09 | 0.89 | 0.000105 |
| *TMEM63A* | -0.16 | -0.16 | -0.09 | -0.39 | -0.02 | 0.89 | 2.35E-05 |
| *MAVS* | -0.30 | -0.10 | -0.13 | -0.10 | -0.17 | 0.89 | 0.003262 |
| *RBM6* | -0.11 | -0.34 | -0.17 | -0.06 | -0.13 | 0.89 | 6.49E-07 |
| *HNRNPUL2* | -0.37 | -0.02 | -0.09 | -0.32 | -0.01 | 0.89 | 0.000451 |
| *TUBGCP5* | -0.15 | 0.00 | -0.07 | -0.52 | -0.07 | 0.89 | 7.22E-08 |
| *GRAMD2B* | -0.10 | -0.11 | -0.22 | -0.29 | -0.09 | 0.89 | 0.000912 |
| *ADAM28* | -0.03 | -0.34 | -0.04 | -0.34 | -0.05 | 0.89 | 0.000212 |
| *CLK2* | -0.25 | -0.12 | -0.13 | -0.23 | -0.07 | 0.89 | 0.01213 |
| *ABCA5* | -0.17 | -0.16 | -0.20 | -0.24 | -0.04 | 0.89 | 0.000174 |
| *ATXN2L* | -0.18 | 0.00 | -0.04 | -0.55 | -0.01 | 0.90 | 0 |
| *IL12RB2* | -0.09 | -0.16 | -0.17 | -0.28 | -0.09 | 0.90 | 0.000367 |
| *RAB11FIP3* | -0.20 | -0.15 | -0.14 | -0.24 | -0.06 | 0.90 | 0.000198 |
| *COLQ* | -0.18 | -0.17 | -0.12 | -0.16 | -0.17 | 0.90 | 0.001241 |
| *ERMP1* | -0.11 | -0.08 | -0.20 | -0.26 | -0.14 | 0.90 | 5.77E-07 |
| *DOPEY2* | -0.13 | -0.07 | 0.00 | -0.45 | -0.13 | 0.90 | 0.006183 |
| *PTPN18* | -0.33 | -0.05 | -0.10 | -0.13 | -0.17 | 0.90 | 0.008685 |
| *SNRK* | -0.49 | -0.02 | -0.11 | -0.04 | -0.12 | 0.90 | 0 |
| *LIG1* | -0.17 | -0.07 | -0.05 | -0.49 | -0.01 | 0.90 | 0 |
| *CHMP7* | -0.06 | -0.09 | -0.05 | -0.55 | -0.05 | 0.90 | 0.000521 |
| *ECHDC2* | -0.13 | -0.16 | -0.16 | -0.18 | -0.15 | 0.90 | 0.02133 |
| *ENOSF1* | -0.06 | -0.22 | -0.22 | -0.25 | -0.04 | 0.90 | 0.000409 |
| *PPP1R13B* | -0.16 | -0.14 | -0.01 | -0.47 | 0.00 | 0.90 | 0.01202 |
| *IARS* | -0.26 | -0.12 | -0.14 | -0.17 | -0.09 | 0.90 | 0.001457 |
| *DET1* | -0.17 | -0.19 | -0.20 | -0.12 | -0.10 | 0.90 | 2.19E-05 |
| *NEMP1* | -0.24 | -0.07 | -0.14 | -0.16 | -0.17 | 0.90 | 0.001271 |
| *PIEZO1* | -0.35 | -0.05 | -0.05 | -0.27 | -0.06 | 0.90 | 6.51E-05 |
| *ZNF266* | -0.10 | -0.26 | -0.12 | -0.14 | -0.16 | 0.90 | 0 |
| *SMARCC2* | -0.29 | -0.14 | -0.13 | -0.06 | -0.16 | 0.90 | 4.38E-05 |
| *ASH1L* | -0.32 | -0.10 | -0.15 | -0.10 | -0.10 | 0.90 | 0 |
| *ICE1* | -0.23 | -0.07 | -0.09 | -0.35 | -0.03 | 0.90 | 3.61E-07 |
| *MTMR1* | -0.46 | -0.01 | -0.03 | -0.21 | -0.07 | 0.90 | 1.44E-07 |
| *GPATCH1* | -0.06 | -0.22 | -0.17 | -0.15 | -0.18 | 0.90 | 0 |
| *SERPINE2* | -0.51 | -0.03 | -0.11 | -0.12 | 0.00 | 0.90 | 0.0298 |
| *SESN1* | -0.04 | -0.28 | -0.16 | -0.27 | -0.01 | 0.90 | 0 |
| *KIAA0753* | -0.29 | -0.13 | -0.04 | -0.30 | -0.01 | 0.90 | 3.85E-05 |
| *URGCP* | -0.20 | -0.16 | -0.08 | -0.28 | -0.03 | 0.90 | 3.23E-05 |
| *ST6GAL1* | -0.46 | -0.03 | -0.03 | -0.14 | -0.11 | 0.90 | 0.002612 |
| *CBR4* | -0.05 | -0.21 | -0.17 | -0.02 | -0.30 | 0.90 | 0 |
| *CBLL1* | -0.24 | -0.08 | -0.10 | -0.14 | -0.20 | 0.90 | 3.54E-06 |
| *ZCCHC11* | -0.16 | -0.14 | -0.20 | -0.20 | -0.07 | 0.90 | 5.56E-06 |
| *DNAJA3* | -0.22 | -0.21 | -0.07 | -0.21 | -0.04 | 0.90 | 0.007297 |
| *DDX42* | -0.38 | 0.00 | -0.08 | -0.20 | -0.09 | 0.90 | 0.002889 |
| *ALDH18A1* | -0.38 | -0.18 | -0.04 | -0.07 | -0.08 | 0.90 | 0 |
| *IL27RA* | -0.19 | -0.05 | -0.04 | -0.37 | -0.09 | 0.90 | 0 |
| *CRTC3* | -0.20 | -0.13 | -0.11 | -0.16 | -0.14 | 0.90 | 0.000272 |
| *PPRC1* | -0.20 | -0.19 | -0.07 | -0.15 | -0.12 | 0.90 | 0.000255 |
| *ARHGEF3* | -0.25 | 0.00 | -0.21 | -0.26 | -0.01 | 0.90 | 0.000146 |
| *ARHGEF9* | -0.14 | -0.01 | -0.14 | -0.27 | -0.18 | 0.90 | 7.22E-08 |
| *RBM39* | 0.00 | -0.24 | -0.22 | -0.19 | -0.07 | 0.90 | 0.00319 |
| *NOL9* | -0.32 | -0.09 | -0.08 | -0.12 | -0.13 | 0.90 | 6.93E-05 |
| *CRNKL1* | -0.12 | -0.16 | -0.11 | -0.03 | -0.32 | 0.90 | 1.59E-06 |
| *IRF3* | -0.35 | -0.09 | -0.05 | -0.08 | -0.16 | 0.90 | 7.50E-06 |
| *MGA* | -0.14 | -0.10 | -0.16 | -0.21 | -0.12 | 0.90 | 0 |
| *IRF8* | -0.12 | -0.17 | -0.03 | -0.39 | -0.02 | 0.90 | 0.000888 |
| *TAF4B* | -0.07 | -0.07 | -0.03 | -0.40 | -0.16 | 0.90 | 0.02245 |
| *CTC1* | -0.18 | -0.14 | -0.07 | -0.34 | -0.01 | 0.90 | 0.04298 |
| *CABIN1* | -0.42 | -0.04 | -0.01 | -0.23 | -0.03 | 0.90 | 0.000634 |
| *HERC1* | -0.45 | -0.06 | -0.10 | -0.10 | -0.02 | 0.90 | 0 |
| *MPHOSPH8* | -0.39 | -0.12 | -0.09 | -0.01 | -0.11 | 0.91 | 0 |
| *BTN3A3* | -0.01 | -0.05 | -0.22 | -0.32 | -0.12 | 0.91 | 0 |
| *RNF34* | -0.22 | -0.21 | -0.10 | -0.07 | -0.12 | 0.91 | 0.01533 |
| *MUM1* | -0.21 | -0.15 | -0.02 | -0.26 | -0.08 | 0.91 | 0.004054 |
| *SERPINF1* | -0.01 | -0.11 | -0.04 | -0.27 | -0.28 | 0.91 | 7.22E-05 |
| *ZNF692* | -0.49 | -0.11 | -0.05 | -0.06 | 0.00 | 0.91 | 0.003497 |
| *TCERG1* | -0.23 | -0.20 | -0.21 | -0.02 | -0.05 | 0.91 | 0 |
| *MDC1* | -0.29 | -0.15 | -0.07 | -0.15 | -0.05 | 0.91 | 7.22E-08 |
| *TLE1* | -0.25 | -0.10 | -0.01 | -0.34 | 0.00 | 0.91 | 7.07E-06 |
| *IKZF1* | -0.26 | -0.03 | -0.07 | -0.20 | -0.15 | 0.91 | 0.000165 |
| *POLG2* | -0.06 | -0.16 | -0.18 | -0.20 | -0.11 | 0.91 | 1.52E-05 |
| *FLNB* | -0.46 | -0.11 | -0.03 | -0.08 | -0.04 | 0.91 | 0.04915 |
| *DMXL1* | -0.09 | -0.25 | -0.17 | -0.05 | -0.14 | 0.91 | 0 |
| *ZNF264* | -0.27 | -0.01 | -0.15 | -0.07 | -0.22 | 0.91 | 0 |
| *DDX31* | -0.21 | -0.18 | -0.03 | -0.22 | -0.06 | 0.91 | 0 |
| *TSPAN3* | -0.04 | -0.05 | -0.03 | -0.52 | -0.08 | 0.91 | 0.009651 |
| *GSAP* | -0.34 | -0.02 | -0.28 | -0.07 | 0.00 | 0.91 | 0.03431 |
| *THOC1* | -0.09 | -0.10 | -0.15 | -0.32 | -0.04 | 0.91 | 0 |
| *ZNF248* | -0.10 | -0.20 | -0.04 | -0.27 | -0.08 | 0.91 | 0.03089 |
| *TAF1C* | -0.22 | -0.19 | -0.08 | -0.18 | -0.03 | 0.91 | 0.02712 |
| *AUTS2* | -0.18 | -0.12 | -0.17 | -0.19 | -0.04 | 0.91 | 0.000645 |
| *PNISR* | -0.06 | -0.25 | -0.20 | -0.04 | -0.14 | 0.91 | 0 |
| *MTHFD1* | -0.15 | -0.10 | -0.20 | -0.22 | -0.02 | 0.91 | 7.22E-07 |
| *COG2* | -0.14 | -0.20 | -0.09 | -0.14 | -0.12 | 0.91 | 0.000712 |
| *SRSF1* | -0.32 | -0.06 | -0.09 | -0.10 | -0.12 | 0.91 | 0.03632 |
| *GPM6B* | -0.16 | -0.12 | -0.06 | -0.06 | -0.28 | 0.91 | 6.28E-06 |
| *MAML1* | -0.39 | -0.03 | -0.04 | -0.16 | -0.07 | 0.91 | 0.008215 |
| *POMT1* | -0.18 | -0.15 | -0.01 | -0.26 | -0.08 | 0.91 | 1.44E-07 |
| *PRRC2B* | -0.40 | -0.02 | -0.05 | -0.15 | -0.06 | 0.91 | 0.001518 |
| *JADE2* | -0.20 | -0.11 | -0.10 | -0.20 | -0.07 | 0.91 | 0 |
| *NUP153* | -0.50 | -0.08 | -0.10 | 0.00 | 0.00 | 0.91 | 0.003189 |
| *PUM1* | -0.33 | -0.02 | -0.12 | -0.12 | -0.08 | 0.91 | 0 |
| *SON* | -0.25 | -0.05 | -0.13 | -0.24 | -0.01 | 0.91 | 4.55E-06 |
| *FUBP1* | -0.25 | -0.08 | -0.18 | -0.06 | -0.11 | 0.91 | 0.000506 |
| *ATR* | -0.16 | -0.08 | -0.14 | -0.26 | -0.04 | 0.91 | 7.22E-08 |
| *MYO9A* | -0.25 | -0.18 | -0.10 | -0.09 | -0.05 | 0.91 | 0.00014 |
| *NFATC3* | -0.16 | -0.08 | -0.19 | -0.15 | -0.09 | 0.91 | 2.68E-05 |
| *LPCAT1* | -0.21 | -0.10 | -0.08 | -0.13 | -0.14 | 0.91 | 0.000904 |
| *ZNF767P* | -0.07 | -0.32 | -0.03 | -0.09 | -0.15 | 0.91 | 0.000848 |
| *TAF11* | -0.07 | -0.06 | -0.18 | -0.25 | -0.12 | 0.91 | 0.0206 |
| *MACF1* | -0.20 | -0.17 | -0.09 | -0.18 | -0.03 | 0.91 | 0.006788 |
| *LMNB2* | -0.29 | -0.02 | -0.08 | -0.17 | -0.10 | 0.91 | 0.001682 |
| *TACC1* | -0.38 | -0.08 | -0.06 | -0.06 | -0.08 | 0.91 | 6.35E-06 |
| *ADNP* | -0.37 | -0.06 | -0.10 | -0.04 | -0.09 | 0.91 | 7.22E-08 |
| *GSPT2* | -0.16 | -0.13 | -0.12 | -0.02 | -0.23 | 0.91 | 7.22E-08 |
| *PLEKHF1* | 0.00 | -0.20 | -0.03 | -0.39 | -0.03 | 0.91 | 3.59E-05 |
| *BTN3A1* | -0.10 | -0.15 | -0.25 | -0.04 | -0.12 | 0.91 | 0.01626 |
| *PCMTD2* | -0.16 | -0.06 | -0.19 | -0.20 | -0.05 | 0.91 | 0.02648 |
| *RNF125* | -0.07 | -0.03 | -0.26 | -0.28 | -0.02 | 0.91 | 0 |
| *CUX2* | -0.17 | -0.13 | -0.01 | -0.24 | -0.10 | 0.91 | 7.29E-06 |
| *ZMYM4* | -0.09 | -0.09 | -0.13 | -0.18 | -0.15 | 0.91 | 0.01789 |
| *QRICH1* | -0.29 | -0.14 | -0.01 | -0.19 | -0.02 | 0.91 | 0.02508 |
| *ARHGEF7* | -0.36 | -0.15 | -0.01 | -0.08 | -0.05 | 0.91 | 0.000107 |
| *POLM* | -0.44 | -0.02 | 0.00 | -0.18 | -0.01 | 0.91 | 1.66E-06 |
| *SPDL1* | -0.04 | -0.12 | -0.19 | -0.22 | -0.07 | 0.91 | 0 |
| *CTR9* | -0.20 | -0.06 | -0.09 | -0.06 | -0.23 | 0.91 | 0.004598 |
| *SEPT6* | -0.06 | -0.08 | -0.16 | -0.20 | -0.16 | 0.91 | 5.51E-05 |
| *NCAPD3* | -0.27 | -0.09 | -0.19 | -0.04 | -0.07 | 0.91 | 0.004806 |
| *ELMO2* | -0.48 | 0.00 | -0.03 | -0.04 | -0.10 | 0.92 | 7.78E-05 |
| *LRRC47* | -0.33 | -0.02 | -0.04 | -0.13 | -0.13 | 0.92 | 0 |
| *FKTN* | -0.17 | -0.12 | -0.08 | -0.16 | -0.11 | 0.92 | 9.09E-06 |
| *AEBP1* | -0.26 | -0.07 | 0.00 | -0.29 | -0.02 | 0.92 | 0.000149 |
| *ERCC5* | -0.17 | -0.13 | -0.08 | -0.20 | -0.04 | 0.92 | 2.87E-05 |
| *INSIG2* | -0.09 | -0.07 | -0.14 | -0.20 | -0.13 | 0.92 | 0.002611 |
| *WDR3* | -0.18 | -0.09 | -0.09 | -0.16 | -0.11 | 0.92 | 1.44E-07 |
| *TRMT1L* | -0.06 | -0.08 | -0.05 | -0.01 | -0.43 | 0.92 | 7.09E-05 |
| *ITGB7* | -0.24 | -0.06 | -0.04 | -0.24 | -0.05 | 0.92 | 5.77E-06 |
| *TBP* | -0.24 | -0.12 | -0.08 | -0.08 | -0.11 | 0.92 | 1.59E-06 |
| *TULP4* | -0.23 | -0.07 | -0.05 | -0.21 | -0.07 | 0.92 | 0.003287 |
| *FUBP3* | -0.15 | -0.09 | -0.17 | -0.13 | -0.10 | 0.92 | 6.28E-06 |
| *ZNF227* | -0.12 | -0.01 | -0.18 | -0.15 | -0.18 | 0.92 | 0.000171 |
| *USP24* | -0.12 | -0.17 | -0.18 | -0.06 | -0.09 | 0.92 | 1.95E-06 |
| *TWNK* | -0.19 | -0.19 | 0.00 | -0.14 | -0.09 | 0.92 | 0.03503 |
| *ATRN* | -0.22 | -0.01 | -0.06 | -0.22 | -0.10 | 0.92 | 0.002946 |
| *MFSD12* | -0.18 | -0.07 | -0.01 | -0.32 | -0.04 | 0.92 | 0.002206 |
| *ZNF589* | -0.22 | -0.09 | -0.01 | -0.22 | -0.08 | 0.92 | 0.001759 |
| *SNRNP70* | -0.33 | -0.01 | 0.00 | -0.20 | -0.08 | 0.92 | 5.79E-05 |
| *DUSP7* | -0.20 | -0.04 | -0.02 | -0.24 | -0.12 | 0.92 | 2.17E-07 |
| *PARP1* | -0.35 | -0.10 | -0.01 | -0.08 | -0.07 | 0.92 | 0.0192 |
| *MOGS* | -0.34 | -0.03 | -0.03 | -0.16 | -0.05 | 0.92 | 2.17E-07 |
| *PPIL2* | -0.25 | -0.08 | -0.02 | -0.25 | -0.01 | 0.92 | 1.44E-07 |
| *HLA-F* | -0.18 | -0.01 | -0.15 | -0.21 | -0.05 | 0.92 | 0.004283 |
| *GSTA1* | -0.15 | -0.24 | -0.04 | -0.03 | -0.15 | 0.92 | 0.005683 |
| *NUP107* | -0.08 | -0.15 | -0.18 | -0.14 | -0.06 | 0.92 | 0.008784 |
| *HNRNPA3* | -0.19 | -0.01 | -0.19 | -0.15 | -0.06 | 0.92 | 0.00012 |
| *DAZAP1* | -0.25 | -0.10 | 0.00 | -0.18 | -0.06 | 0.92 | 0.000832 |
| *CKAP5* | -0.26 | -0.06 | -0.05 | -0.08 | -0.14 | 0.92 | 0.000128 |
| *CEP192* | -0.24 | -0.01 | -0.14 | -0.10 | -0.11 | 0.92 | 3.89E-05 |
| *METTL17* | -0.26 | -0.05 | -0.03 | -0.19 | -0.06 | 0.92 | 7.22E-08 |
| *SLC35C2* | -0.30 | -0.07 | -0.01 | -0.06 | -0.14 | 0.92 | 4.68E-05 |
| *HSPH1* | -0.16 | -0.13 | 0.00 | -0.24 | -0.06 | 0.92 | 5.59E-05 |
| *USPL1* | -0.07 | -0.18 | -0.13 | -0.07 | -0.14 | 0.92 | 1.48E-05 |
| *HMBOX1* | -0.02 | -0.13 | -0.04 | -0.33 | -0.07 | 0.92 | 0.04637 |
| *ACACB* | -0.17 | -0.12 | 0.00 | -0.25 | -0.04 | 0.92 | 0 |
| *ATM* | -0.16 | -0.12 | -0.21 | 0.00 | -0.10 | 0.92 | 0.007097 |
| *MTRF1* | -0.05 | -0.17 | -0.08 | -0.26 | -0.02 | 0.92 | 4.41E-05 |
| *TRAF3* | -0.17 | -0.03 | 0.00 | -0.30 | -0.08 | 0.92 | 0.01477 |
| *WEE1* | -0.10 | -0.18 | -0.20 | -0.04 | -0.06 | 0.92 | 0.0314 |
| *PPP6R1* | -0.22 | -0.13 | -0.03 | -0.18 | -0.02 | 0.92 | 0.03372 |
| *TASP1* | -0.08 | -0.02 | -0.12 | -0.35 | -0.01 | 0.92 | 0.000795 |
| *C2CD2* | -0.14 | -0.12 | -0.04 | -0.12 | -0.17 | 0.92 | 0.0134 |
| *GABBR1* | -0.23 | -0.03 | -0.15 | -0.06 | -0.09 | 0.92 | 0.01327 |
| *DHX15* | -0.13 | -0.11 | -0.19 | -0.10 | -0.04 | 0.92 | 0 |
| *DYNC1H1* | -0.42 | -0.01 | -0.02 | -0.12 | -0.01 | 0.92 | 0.001354 |
| *TFIP11* | -0.15 | -0.13 | -0.05 | -0.02 | -0.22 | 0.92 | 0 |
| *MON1B* | -0.16 | -0.15 | -0.10 | -0.03 | -0.13 | 0.93 | 0.02651 |
| *LRIG2* | -0.24 | -0.06 | -0.10 | -0.14 | -0.02 | 0.93 | 0.01014 |
| *SDCCAG3* | -0.15 | -0.16 | -0.09 | -0.07 | -0.09 | 0.93 | 0.002095 |
| *TRIM68* | -0.16 | -0.14 | -0.13 | -0.08 | -0.05 | 0.93 | 0.04245 |
| *FAM160B2* | -0.24 | -0.08 | -0.01 | -0.22 | -0.01 | 0.93 | 0.009206 |
| *EIF3A* | -0.13 | -0.02 | -0.08 | -0.29 | -0.03 | 0.93 | 0.000126 |
| *HNRNPA2B1* | -0.15 | -0.07 | -0.17 | -0.15 | -0.01 | 0.93 | 0.02067 |
| *DUSP6* | -0.17 | -0.01 | -0.15 | -0.09 | -0.14 | 0.93 | 0.000539 |
| *WDR6* | -0.33 | -0.06 | -0.01 | -0.13 | -0.03 | 0.93 | 8.73E-05 |
| *ZNF814* | -0.14 | -0.11 | -0.05 | -0.11 | -0.13 | 0.93 | 1.44E-07 |
| *SREK1* | -0.25 | -0.01 | -0.10 | -0.14 | -0.05 | 0.93 | 0.02188 |
| *DLG5* | -0.04 | -0.22 | -0.18 | -0.03 | -0.07 | 0.93 | 0 |
| *DYRK1B* | -0.24 | -0.02 | -0.01 | -0.23 | -0.04 | 0.93 | 0.01637 |
| *ZSCAN18* | -0.12 | -0.10 | -0.07 | -0.01 | -0.25 | 0.93 | 6.03E-05 |
| *KIR3DL1* | -0.08 | -0.25 | -0.11 | -0.08 | -0.02 | 0.93 | 0.000261 |
| *SLC9A3R1* | -0.23 | -0.14 | -0.05 | -0.03 | -0.09 | 0.93 | 7.22E-08 |
| *CHST12* | -0.09 | -0.01 | -0.20 | -0.16 | -0.07 | 0.93 | 0.000224 |
| *ZC3H4* | -0.42 | -0.03 | 0.00 | -0.04 | -0.05 | 0.93 | 0.01536 |
| *FARSA* | -0.18 | -0.07 | -0.03 | -0.12 | -0.14 | 0.93 | 0 |
| *REV1* | -0.20 | -0.15 | -0.06 | -0.07 | -0.05 | 0.93 | 0.02651 |
| *SEPT2* | -0.22 | -0.15 | -0.04 | -0.01 | -0.10 | 0.93 | 1.76E-05 |
| *TTC28* | -0.18 | -0.14 | -0.07 | -0.06 | -0.07 | 0.93 | 0.001024 |
| *TM2D3* | -0.13 | -0.02 | -0.13 | -0.10 | -0.14 | 0.93 | 0.004231 |
| *MMS19* | -0.22 | -0.14 | -0.02 | -0.07 | -0.07 | 0.93 | 0.000322 |
| *ROBO3* | -0.15 | -0.13 | -0.11 | -0.11 | -0.02 | 0.93 | 0.004048 |
| *KIR2DL3* | -0.19 | 0.00 | -0.01 | -0.28 | -0.04 | 0.93 | 0.000719 |
| *POLR2B* | -0.14 | -0.11 | -0.16 | -0.03 | -0.07 | 0.93 | 0 |
| *KDM5C* | -0.32 | 0.00 | -0.02 | -0.11 | -0.05 | 0.93 | 0.001685 |
| *CAPRIN2* | -0.13 | -0.06 | -0.06 | -0.22 | -0.03 | 0.93 | 1.44E-07 |
| *RNF4* | -0.26 | -0.05 | -0.10 | -0.06 | -0.02 | 0.93 | 0.008758 |
| *TBC1D4* | -0.05 | -0.03 | -0.20 | -0.17 | -0.04 | 0.93 | 0.0009 |
| *MINDY1* | -0.32 | -0.05 | -0.06 | 0.00 | -0.06 | 0.93 | 2.17E-07 |
| *PIP4K2C* | -0.16 | -0.12 | -0.05 | -0.06 | -0.09 | 0.93 | 7.22E-07 |
| *C2CD5* | -0.23 | -0.04 | -0.18 | -0.01 | -0.02 | 0.93 | 0.0478 |
| *ARGLU1* | -0.05 | -0.12 | -0.14 | -0.06 | -0.11 | 0.93 | 0 |
| *ADAT1* | -0.19 | -0.05 | -0.09 | -0.07 | -0.08 | 0.93 | 0 |
| *SP140L* | -0.20 | -0.11 | -0.01 | -0.16 | -0.01 | 0.93 | 0.000675 |
| *MBD1* | -0.23 | -0.01 | -0.02 | -0.18 | -0.05 | 0.93 | 0.000257 |
| *ZNF274* | -0.19 | -0.18 | -0.02 | -0.10 | 0.00 | 0.93 | 0.03625 |
| *ZNHIT6* | -0.21 | -0.06 | -0.11 | -0.10 | -0.01 | 0.94 | 0.01371 |
| *LSG1* | -0.10 | -0.11 | -0.08 | -0.12 | -0.06 | 0.94 | 1.00E-04 |
| *MECP2* | -0.25 | -0.02 | -0.04 | -0.11 | -0.05 | 0.94 | 0.02226 |
| *DENND4A* | -0.19 | 0.00 | -0.18 | -0.01 | -0.08 | 0.94 | 0.002766 |
| *ARRB1* | -0.19 | -0.01 | -0.01 | -0.13 | -0.13 | 0.94 | 0 |
| *PGRMC2* | -0.02 | -0.02 | -0.14 | -0.24 | -0.05 | 0.94 | 0.02047 |
| *HEATR1* | -0.13 | -0.09 | -0.15 | -0.05 | -0.04 | 0.94 | 0.02708 |
| *ASB1* | -0.17 | -0.04 | -0.01 | -0.24 | -0.01 | 0.94 | 0.02979 |
| *KIF13B* | -0.22 | -0.09 | -0.06 | -0.05 | -0.03 | 0.94 | 0.01607 |
| *WDR19* | -0.15 | -0.13 | -0.02 | -0.12 | -0.02 | 0.94 | 0.001631 |
| *BPTF* | -0.20 | -0.08 | -0.11 | -0.03 | -0.04 | 0.94 | 0.02132 |
| *CHD4* | -0.12 | -0.01 | -0.01 | -0.20 | -0.10 | 0.94 | 0.007014 |
| *CD81* | -0.15 | -0.06 | -0.10 | -0.09 | -0.02 | 0.94 | 0.003379 |
| *KDM2A* | -0.23 | -0.08 | -0.01 | -0.08 | -0.04 | 0.94 | 0.000557 |
| *MCCC2* | -0.20 | 0.00 | -0.04 | -0.10 | -0.09 | 0.94 | 0.008123 |
| *CUL4A* | -0.13 | -0.06 | -0.09 | -0.08 | -0.07 | 0.94 | 0.03676 |
| *HMGCS1* | -0.14 | -0.04 | -0.10 | -0.01 | -0.13 | 0.94 | 0.001322 |
| *TSC2* | -0.23 | -0.07 | 0.00 | -0.06 | -0.06 | 0.94 | 0.04434 |
| *TTF2* | -0.23 | -0.06 | -0.09 | 0.00 | -0.03 | 0.94 | 0.001964 |
| *ABHD17B* | -0.32 | -0.03 | -0.02 | -0.01 | -0.04 | 0.94 | 0.000691 |
| *U2SURP* | 0.00 | -0.06 | -0.21 | -0.10 | -0.05 | 0.94 | 1.41E-05 |
| *LARS2* | -0.20 | -0.04 | -0.08 | -0.04 | -0.05 | 0.94 | 4.95E-05 |
| *MAP3K12* | -0.21 | -0.04 | -0.02 | -0.13 | 0.00 | 0.95 | 0.01111 |
| *C2CD2L* | -0.18 | -0.01 | -0.04 | -0.15 | -0.02 | 0.95 | 0.01573 |
| *ABAT* | -0.18 | -0.05 | -0.13 | -0.01 | -0.03 | 0.95 | 0.04024 |
| *XPO7* | -0.11 | -0.01 | -0.10 | -0.06 | -0.11 | 0.95 | 0.0015 |
| *YTHDF1* | -0.22 | -0.10 | -0.05 | 0.00 | -0.04 | 0.95 | 0.003467 |
| *ANKRD10* | -0.22 | -0.08 | -0.02 | -0.02 | -0.04 | 0.95 | 0.01492 |
| *NSD2* | -0.24 | -0.05 | -0.04 | -0.01 | -0.03 | 0.95 | 0.000485 |
| *CROCCP2* | -0.02 | -0.13 | -0.07 | -0.12 | -0.01 | 0.95 | 0.01254 |
| *GMEB2* | -0.22 | -0.02 | -0.02 | -0.09 | 0.00 | 0.95 | 0.002518 |
| *LINS1* | -0.04 | -0.14 | -0.16 | -0.01 | -0.01 | 0.95 | 0.02843 |
| *ENO3* | -0.08 | -0.05 | -0.01 | -0.16 | -0.05 | 0.95 | 0.01524 |
| *MZF1* | -0.23 | -0.05 | -0.01 | -0.03 | -0.03 | 0.95 | 0.02559 |
| *UPF3B* | 0.00 | -0.05 | -0.02 | -0.16 | -0.06 | 0.96 | 0.005026 |
| *CNDP2* | -0.18 | -0.04 | -0.01 | -0.02 | -0.02 | 0.96 | 0.02957 |

Genes were ranked according to the fold change. LogFC: base 2 log of Fold-change; AveLogFC: average LogFC; FDR: False Discovery Rate. VTE: venous thromboembolism; PAOD: peripheral arterial obstructive disease; AMI: acute myocardial infarction; CS: cardioembolic stroke; IS: ischemic stroke
