# Supplementary material for: Identification of common and divergent gene expression signatures in patients with venous and arterial thrombosis using data from public repositories
Source: PLoS One. 2020 Aug 11;15(8):e0235501. doi: 10.1371/journal.pone.0235501 (PMC7418995; doi:10.1371/journal.pone.0235501)
Supplement: S2 Table — (DOCX) [file pone.0235501.s002.docx]

**Supplementary table 2.** Top differentially expressed genes identified in the meta-analysis of studies involving **acute** CVD

|  | **Fold-change in individual studies** (LogFC) | | **Meta-analysis results** | | **Main biological process** |
| --- | --- | --- | --- | --- | --- |
| **Genes** | **AMI** | **CS** | **Ave**  **LogFC** | **FDR** |  |
| **Up-regulated genes** | | | | |  |
| *MCEMP1* | 0.70 | 1.37 | 1.03 | <0.0001 | Imm Reg |
| *ANXA3* | 0.87 | 0.97 | 0.92 | <0.0001 | Hemostasis |
| *INSC* | 0.47 | 1.33 | 0.89 | <0.0001 | Cell Prolif |
| *ANKRD22* | 0.05 | 1.71 | 0.88 | <0.0001 | Imm Reg |
| *MMP9* | 0.62 | 1.14 | 0.88 | <0.0001 | Cell Maint |
| *SLC26A8* | 0.38 | 1.31 | 0.84 | <0.0001 | Cell Prolif |
| *BMX* | 0.29 | 1.37 | 0.83 | <0.0001 | Cell Prolif |
| *S100A12* | 0.80 | 0.84 | 0.82 | <0.0001 | Innate Imm |
| *OLAH* | 0.16 | 1.44 | 0.80 | <0.0001 | Lipid metabolism |
| *FOLR3* | 0.68 | 0.90 | 0.79 | <0.0001 | Cell Maint |
| **Down-regulated genes** | | | | |  |
| *PDZK1IP1* | -0.15 | -1.15 | -0.65 | <0.0001 | Cell Prolif |
| *PF4V1* | -0.62 | -0.43 | -0.52 | <0.0001 | Cell Prolif |
| *DDX11L1* | -0.24 | -0.63 | -0.44 | <0.0001 | Unknown |
| *PAX8-AS1* | -0.04 | -0.74 | -0.39 | <0.0001 | Cell Prolif |
| *FAM118A* | -0.08 | -0.69 | -0.38 | <0.0001 | Unknown |
| *KLF1* | -0.03 | -0.68 | -0.36 | <0.0001 | Cell Prolif |
| *EIF1AY* | -0.57 | -0.14 | -0.36 | <0.0001 | Unknown |
| *FAXDC2* | -0.12 | -0.55 | -0.33 | <0.0001 | Unknown |
| *HBZ* | -0.25 | -0.39 | -0.32 | <0.0001 | Cell Prolif |
| *TXLNGY* | -0.31 | -0.53 | -0.08 | <0,0001 | Unknown |

Genes were ranked according to the fold change. LogFC: base 2 log of Fold-change; AveLogFC: average LogFC. FDR: False Discovery Rate. Innate Imm: innate immunity; Imm Reg: Immune regulation; Cell Maint: cell maintenance; Cell Prolif: cell proliferation; Gene expr: gene expression; T cell dev: T cell development.
