# Supplementary material for: Identification of common and divergent gene expression signatures in patients with venous and arterial thrombosis using data from public repositories
Source: PLoS One. 2020 Aug 11;15(8):e0235501. doi: 10.1371/journal.pone.0235501 (PMC7418995; doi:10.1371/journal.pone.0235501)
Supplement: S3 Table — (DOCX) [file pone.0235501.s003.docx]

**Supplementary table 3**. Top differentially expressed genes identified in the meta-analysis of studies involving **chronic** CVD

|  | **Fold-change in individual studies** (LogFC) | | **Meta-analysis results** | | **Biological process** (main) |
| --- | --- | --- | --- | --- | --- |
| **Genes** | **IS** | **PAOD** | **Ave**  **LogFC** | **FDR** |  |
| **Up-regulated genes** | | | | |  |
| *G0S2* | 2.29 | 1.96 | 2.13 | <0.0001 | Apoptosis |
| *CXCL8* | 2.65 | 1.58 | 2.12 | <0.0001 | Innate Imm |
| *PTGS2* | 1.76 | 1.46 | 1.61 | <0.0001 | Imm Reg |
| *IL1B* | 1.55 | 0.99 | 1.27 | <0.0001 | Innate Imm |
| *NR4A2* | 1.50 | 0.95 | 1.22 | <0.0001 | Cell Prolif |
| *CCL20* | 1.51 | 0.67 | 1.09 | <0.0001 | Cell Prolif |
| *PPP1R15A* | 1.15 | 0.93 | 1.04 | <0.0001 | Imm Reg |
| *EGR3* | 0.78 | 1.19 | 0.99 | <0.0001 | Imm Reg |
| *EREG* | 1.13 | 0.76 | 0.95 | <0.0001 | Imm Reg |
| *EGR2* | 0.97 | 0.91 | 0.94 | <0.0001 | Imm Reg |
| **Down-regulated genes** | | | | |  |
| *TNFRSF17* | -1.03 | -0.47 | -0.75 | <0.0001 | Imm Reg |
| *JCHAIN* | -0.65 | -0.33 | -0.49 | <0.0001 | Unknown |
| *TAPT1-AS1* | -0.44 | -0.49 | -0.46 | <0.0001 | Unknown |
| *FTX* | -0.46 | -0.39 | -0.43 | <0.0001 | Cell Prolif |
| *TRMT13* | -0.41 | -0.44 | -0.42 | <0.0001 | Unknown |
| *EIF3C* | -0.44 | -0.36 | -0.40 | <0.0001 | Cell Prolif |
| *ZNF302* | -0.46 | -0.33 | -0.40 | <0.0001 | Unknown |
| *ZFP3* | -0.38 | -0.34 | -0.36 | <0.0001 | Unknown |
| *TCL1A* | -0.36 | -0.34 | -0.35 | <0.0001 | T cell dev |

Genes were ranked according to the fold change. LogFC: base 2 log of Fold-change; AveLogFC: average LogFC; FDR: False Discovery Rate. Innate Imm: innate immunity; Imm Reg: Immune regulation; Cell Maint: cell maintenance; Cell Prolif: cell proliferation; Gene expr: gene expression; T cell dev: T cell development.
