# Supplementary material for: Identification of common and divergent gene expression signatures in patients with venous and arterial thrombosis using data from public repositories
Source: PLoS One. 2020 Aug 11;15(8):e0235501. doi: 10.1371/journal.pone.0235501 (PMC7418995; doi:10.1371/journal.pone.0235501)
Supplement: S4 Table — (DOCX) [file pone.0235501.s004.docx]

**Supplementary table 4**. Divergently expressed genes in CVD and VTE

| Genes that are up-regulated in VTE and down-regulated in CVD (**VTE>CVD**) | Genes that are down-regulated in VTE and up-regulated in CVD (**CVD>VTE**) |
| --- | --- |
| *PTCD2* | *FFAR2* |
| *MRPL22* | *MMP9* |
| *MRPS33* | *ACSL1* |
| *NDUFB2* | *KIAA1324* |
| *SRRD* | *PELI1* |
| *PSMC6* | *ANPEP* |
| *VRK1* | *BACH1* |
| *MRPS31* | *ICAM1* |
| *EIF3E* | *CHMP1B* |
| *CLC* | *PPIF* |
| *TMEM106B* | *PACSIN2* |
| *SMURF2* | *MCL1* |
| *CLEC2B* | *PELI2* |
| *RWDD1* | *SLC6A6* |
| *COX7A2* | *AOC3* |
| *GZMK* | *ITGAX* |
| *TTC33* | *TAB2* |
| *CRBN* | *DAZAP2* |
| *SLC25A36* | *TRIM28* |
| *BBX* | *EHD1* |
| *SNX4* | *RAB21* |
| *TXNDC9* | *PADI4* |
| *CAPZA1* | *CIC* |
| *CD48* | *RFX2* |
| *ZNF430* | *PIM2* |
| *VAMP8* | *ANAPC2* |
| *PYROXD1* | *GRK2* |
| *KLHDC10* | *MAPKAPK2* |
| *LGALS3* | *SORBS3* |
| *C12orf29* | *SF3B2* |
| *ANXA1* | *TSPYL2* |
| *RPL35* | *CBX4* |
| *RPS24* | *SF3A1* |
| *COPS4* | *VAMP2* |
| *PPA2* | *RARA* |
| *SCAF11* | *CAPN15* |
| *PIGK* | *JUND* |
| *SNRPG* | *ALPL* |
| *GZMA* | *RHOB* |
| *NKAPD1* | *CD5* |
| *SREK1IP1* | *MEF2D* |
| *CHD1* | *TET3* |
| *SLC26A2* | *CNN2* |
| *GTPBP8* | *TSC22D3* |
| *ZNF148* | *SRSF5* |
| *DNAJB14* | *H1FX* |
| *UQCRQ* | *TAF6* |
| *ZNF22* | *ARHGDIA* |
| *ATP5F1C* | *MAP4K2* |
| *PTER* | *RAB5C* |
| *ZNF91* | *LSM14B* |
| *NAT1* | *TLN1* |
| *RPS21* | *GRAP* |
| *ZWILCH* |  |
| *P2RY14* |  |
| *ZNF654* |  |
| *RPS27* |  |
| *SUZ12* |  |
| *AP1S2* |  |
| *NDUFB1* |  |
| *RPS27L* |  |
| *ZNF292* |  |
| *NDUFA4* |  |
| *UBL5* |  |
| *DENND1B* |  |
| *RPS29* |  |
| *MCTS1* |  |
| *ZNF271P* |  |
| *GTF2H5* |  |
| *AKR1C3* |  |
| *LAIR2* |  |
