# Supplementary material for: Identification of common and divergent gene expression signatures in patients with venous and arterial thrombosis using data from public repositories
Source: PLoS One. 2020 Aug 11;15(8):e0235501. doi: 10.1371/journal.pone.0235501 (PMC7418995; doi:10.1371/journal.pone.0235501)
Supplement: S1 Fig — Correlation of gene expression changes studies of CVD whose samples were collected in the acute (a) or chronic (b) phase of their disease courses. Ischemic stroke (IS), Peripheral arterial occlusive disease (PAOD), Acute myocardial infarction (AMI) and Cardioembolic stroke (CS). Pairwise correlation scatter plots are in the lower triangle boxes. The upper triangle boxes show Pearson correlation coefficients (R) of log2 fold changes for all 472 differentially expressed genes identified in the meta-analysis of all 5 studies. (DOCX) [file pone.0235501.s006.docx]

**Supplementary figure 1.** Correlation between VTE and CVD (acute and chronic)


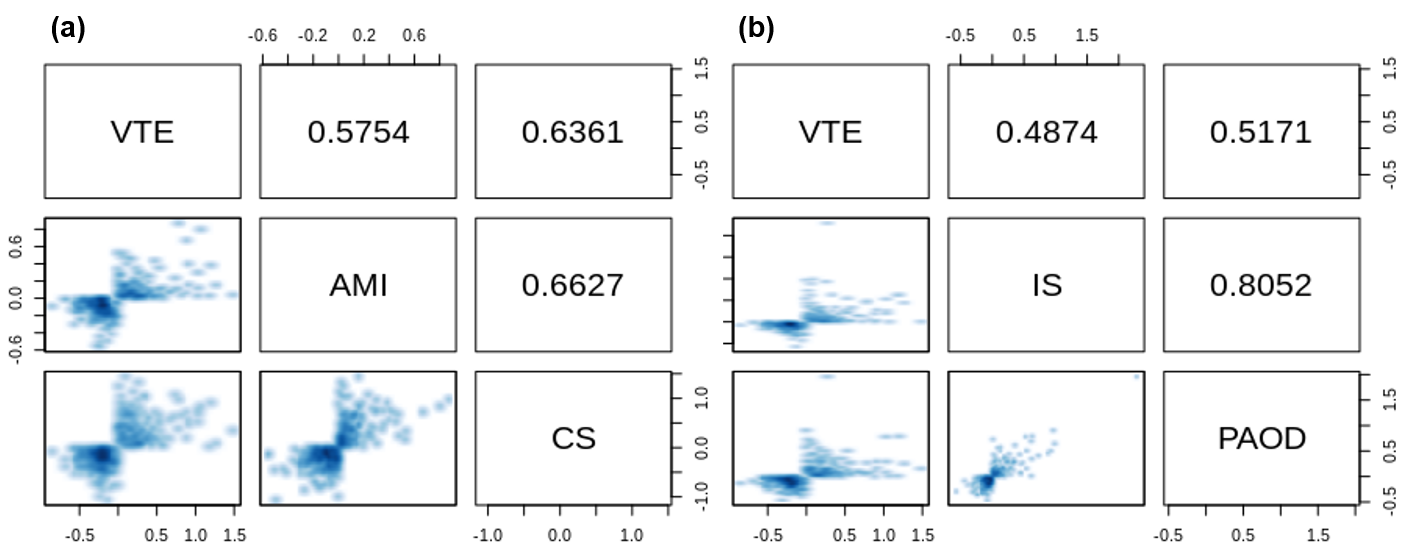


**Supplementary figure 1.** Correlation of gene expression changes studies of CVD whose samples were collected in the acute (a) or chronic (b) phase of their disease courses. Ischemic stroke (IS), Peripheral arterial occlusive disease (PAOD), Acute myocardial infarction (AMI) and Cardioembolic stroke (CS). Pairwise correlation scatter plots are in the lower triangle boxes. The upper triangle boxes show Pearson correlation coefficients (R) of log2 fold changes for all 472 differentially expressed genes identified in the meta-analysis of all 5 studies.
